# How Do Haloarchaea Synthesize Aromatic Amino Acids?

**DOI:** 10.1371/journal.pone.0107475

**Published:** 2014-09-12

**Authors:** Miriam Kolog Gulko, Mike Dyall-Smith, Orland Gonzalez, Dieter Oesterhelt

**Affiliations:** 1 Department of Membrane Biochemistry, Max-Planck-Institute of Biochemistry, Martinsried, Germany; 2 School of Biomedical Sciences, Charles Sturt University, Wagga Wagga, Australia; 3 Teaching and Research Unit Bioinformatics, Institut of Informatik Ludwig-Maximilians-University, Munich, Germany; Berlin Institute of Technology, Germany

## Abstract

Genomic analysis of *H. salinarum* indicated that the *de novo* pathway for aromatic amino acid (AroAA) biosynthesis does not follow the classical pathway but begins from non-classical precursors, as is the case for *M. jannaschii*. The first two steps in the pathway were predicted to be carried out by genes OE1472F and OE1475F, while the 3^rd^ step follows the canonical pathway involving gene OE1477R. The functions of these genes and their products were tested by biochemical and genetic methods. In this study, we provide evidence that supports the role of proteins OE1472F and OE1475F catalyzing consecutive enzymatic reactions leading to the production of 3-dehydroquinate (DHQ), after which AroAA production proceeds via the canonical pathway starting with the formation of DHS (dehydroshikimate), catalyzed by the product of ORF OE1477R. Nutritional requirements and AroAA uptake studies of the mutants gave results that were consistent with the proposed roles of these ORFs in AroAA biosynthesis. DNA microarray data indicated that the 13 genes of the canonical pathway appear to be utilised for AroAA biosynthesis in *H. salinarum*, as they are differentially expressed when cells are grown in medium lacking AroAA.

## Introduction

Eukaryotes and bacteria synthesize aromatic amino acids (AroAA) via the shikimate pathway, which in the well-studied *E.coli* system comprises 17 different enzymes [1], [2]. In haloarchaea, 13 recognizable homologs of the shikimate pathway enzymes can be identified, and these cover all of the later steps necessary to convert 3-dehydroquinate (DHQ) to AroAA. However, homologues for the initial reactions responsible for the biosynthesis of DHQ are not present, suggesting that a non-canonical pathway for AroAA biosynthesis is used in haloarchaea. This is supported by other lines of circumstantial evidence.

Firstly, the canonical precursor for AroAA biosynthesis is erythrose-4-phosphate (E-4-P), which is a product of the pentose phosphate pathway (PPP). However, this pathway does not appear to be present in the archaeal domain, and only some orthologs are present in varying degrees [3]. In halophilic archaea, only two enzymes related to the pentose phosphate pathway have been proposed [3]–[5], and unless there is an alternative pathway for the production of E-4-P, this compound is not available for the conventional AroAA pathway in haloarchaea.

**Figure 1:**
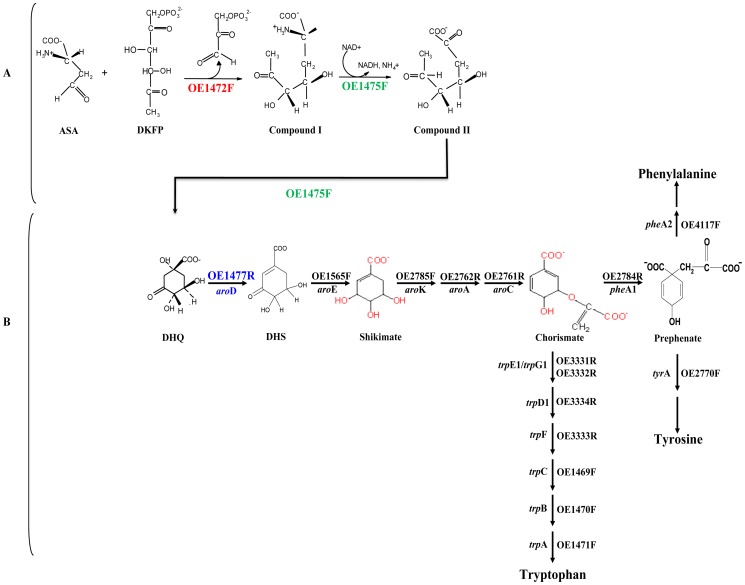
Proposed pathway for the biosynthesis of AroAA in *H*. *salinarum*, based on the pathway described for *M*. *jannaschii*. **A**, The initial steps in the de novo pathway were proposed according to White [6]. Note that no transaldolase reaction with ASA+DKFP was detected in this study, whereas the detected aldolase activity of OE1472F suggests that in *H*. *salinarum* the precursor might be F-1,6-P rather than DKFP. For details see Fig 10 and discussion. **B**, Downstream to DHQ, the canonical pathway is followed. Protein homologs found in *H*. *salinarum* are indicated above (or to the right of) the arrows, and the genes names are indicated below (or to the left). ASA-L-aspartate semialdehyde, DKFP-6-deoxy-5-ketofructose 1-phosphate, DHQ-dehydroquinate, DHS- dehydroshikimate.

Secondly, the methanogenic archaeon *M*. *jannaschii* has been shown to use an alternative pathway of DHQ biosynthesis [6], carried out by enzymes that are unrelated to those of the classical pathway. Methanogens and haloarchaea are both members of the phylum *Euryarchaeota*, and phylogenetic reconstructions frequently show haloarchaea originating from within methanogen clades [7], [8]. Consistent with this phylogenetic relationship, close homologues of *M*. *jannaschii* genes MJ0400 and MJ1249, which specify enzymes at he beginning of AroAA pathway, are encoded in all available haloarchaeal genomes (40–47% aa similarity; 16 genomes according to NCBI, October 2012). In *H*. *salinarum*, these are ORFs OE1472F and OE1475F, which we propose as candidates for the first two steps in the biosynthesis of AroAA (Fig. 1A).

Thirdly, homologues of all 13 genes of the classical pathway that are needed to convert DHQ to AroAA are present in all sequenced haloarchaeal genomes (Fig. 1B, www.halolex.mpg.de). This supports the idea that downstream of DHQ the synthesis of AroAA follows the canonical pathway, as is also the case with *M*. *jannaschii*.

In the alternative pathway suggested by White for *M*. *jannaschii* [6], the first step is a transaldolase (TA) reaction between 6-deoxy-5-ketofructose 1-phosphate (DKFP) and aspartate semialdehyde (ASA). In *H*. *salinarum*, this step is proposed to be carried out by the enzyme specified by ORF OE1472F (homologue of MJ0400). Hydroxpyruvaldehyde phosphate (HPAP) would be released and compound I formed. In the second stage, DHQ is proposed to be formed by oxidative deamination and cyclization catalyzed by the enzyme specified by ORF OE1475F (homologue of MJ1249). From this stage onwards the canonical pathway can be used for the biosynthesis of AroAA (Fig. 1B).

In this study, we tested the hypothesis that haloarchaea use the same reaction pathway for AroAA synthesis as shown experimentally for *M*. *jannaschii* by White [6]. For this, the model haloarchaeon *H*. *salinarum* was analysed using both *in vivo* and *in vitro* strategies, including targeted mutations of the proposed first two genes, nutrient requirements, phenotypes, AroAA uptake assays, enzyme activities of the purified gene products and global examination of AroAA-related genes using a genome-wide DNA microarray. We provide evidence that the proteins OE1472F and OE1475F do specify enzymes that provide DHQ to feed synthesis of AroAA in haloarchaea. For brevity, we will refer to the hypothesized AroAA pathway in *H.salinarum* as the **proposed pathway** (i.e. a non-canonical pathway like that of *M. jannaschii*).

## Results

### Gene selection and construction of knock- out mutants

**Figure 2:**
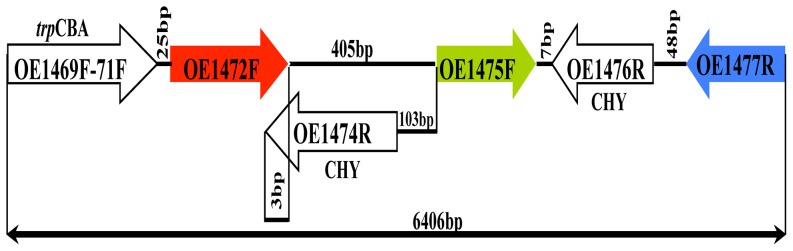
Schematic representation of ORFs OE1472F, OE1475F and OE1477R in the *H. salinarum* R1 genome (accession number AM774415.1), and their relation to surrounding genes. CHY- conserved hypothetical protein. Arrows show the relative positions and orientations of ORFs (but are not drawn to scale). Coordinates 230688–237094 bp.

Based on available data, and the experimentally validated pathway of AroAA biosynthesis in *M*. *jannaschii* [6], the first three steps would be catalyzed by ORFs OE1472F, OE1475F and OE1477R (*aro*D), respectively (Fig 1). A diagram of these genes and their relation to surrounding genes is shown in figure 2. In-frame deletions of OE1472F, OE1475F and OE1477R should convert *H*. *salinarum* to aromatic amino acid auxotrophy, confirming the role of these ORFs in the pathway.

**Figure 3:**
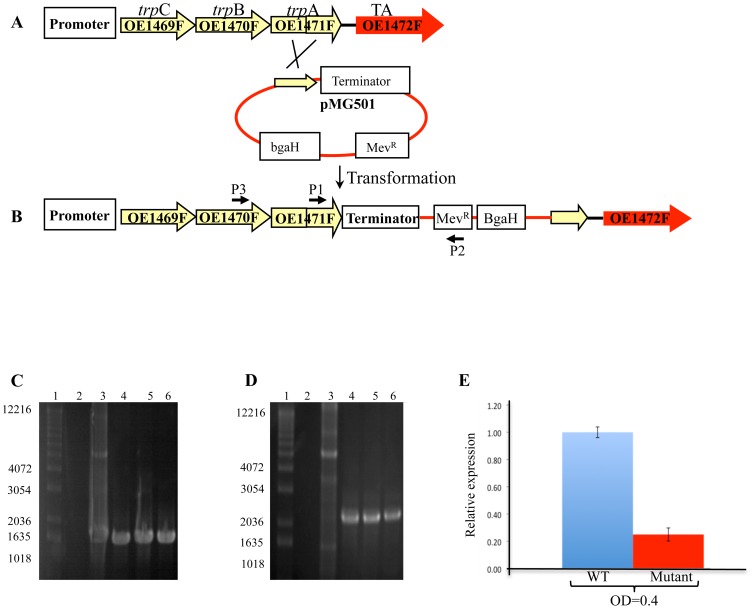
Schematic representations of a single cross-over event between the chromosomal DNA of *H. salinarum* R1 and plasmid pMG501. P1,P2 and P3 and their associated arrows indicate the relative positions and orientations of the primers used for PCR. After in-frame integration of plasmid pMG501, PCR was expected to generate a product in transformed cells but not for the WT. **A**, Illustration of WT *H. salinarum* genotype. **B**, illustration of the mutant genotype after incorporation of plasmid pMG501. **C** and **D**, Agarose gel electrophoresis of PCR products using primers P1 and P2, and primers P3 and P2, respectively. Lane 1: MW markers (in bp), lane2: Chromosomal DNA of R1, lane 3: plasmid pMG501 (control), lanes 4–6- transformed colonies. **E**, Relative expression of OE1472F transcription in mutant *Stop*OE1472F (OE1471F::pMG501) grown in synthetic medium without AroAA to OD_600nm_ = 0.4 and 1.0. Results were obtained using RT-qPCR. See table S7 in file S1 for details of the primers used.

An in-frame deletion strain of *aro*D (OE1477R) was readily obtained, but similar attempts to delete ORFs OE1472F and OE1475F were repeatedly unsuccessful. In the latter cases, transformants invariably reverted to the wild type (WT) rather than to a deletion genotype (data not shown). Instead of complete deletions of OE1472F and OE1475F, attempts were then made to reduce their expression by insertional mutagenesis. As shown in Fig. 3A–B, a plasmid-borne terminator sequence was inserted just upstream of ORF OE1472F and immediately downstream of the adjacent *trp*A, creating a stable mutant OE1471F::pMG501 (*Stop*OE1472F). This construct was confirmed by both Southern blot (Fig. S1A in File S1) and PCR (Fig. 3C). Although pMG501 did not contain an origin of replication for haloarchaea, an additional PCR was performed to exclude the possibility that pMG501 could survive in R1 without integration into the chromosome. As expected, WT or plasmid DNA did not produce an amplification product (Fig. 3D). Transcription of ORF OE1472F in the insertion mutant *Stop*OE1472F, was measured by RT-PCR and found to be 44-fold less in the mutant compared to WT (Fig. 3E), indicating that the terminator-containing plasmid insertion had indeed reduced transcription of the target gene. To check the effect of the terminator-containing plasmid on the expression of nearby ORFs in the *trp*CBA operon, namely ORFs OE1469F, OE1470F and OE1471F, their expression was examined by RT-PCR. While no change in the expression of ORF OE1469F was observed, both OE1470F and OE1471F were up regulated by 2–3 fold (Fig. S2 in File S1). Increased transcription levels were also observed for ORFs of the *trp*D_1_FE_1_G*1* operon (1.5–5 fold up regulation) (Fig S2B in file S1).

**Figure 4:**
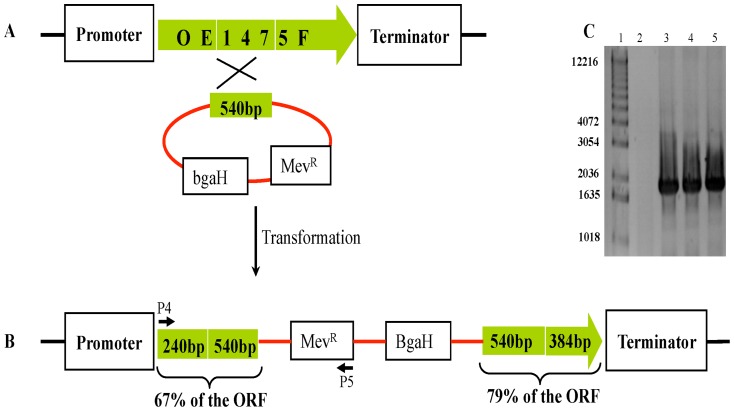
Schematic representation of a single cross-over event between plasmid pMG601 and *H. salinarum* R1. **A** and **B**, illustration of WT and the mutant genotype after incorporation of plasmid pMG501, respectively, **C**, Agarose gel of PCR products from chromosomal DNA of strain R1 (lane 2), and chromosomal DNA from 3 *Bga*H^+^ colonies after integration of pMG601 into R1 (lane 3–5), MW marker is shown in lane 1 (in bp).

An insertion mutant of ORF OE1475F was obtained by homologous integration of a plasmid containing only the central part of this ORF. In this construct, (OE1475F::pMG601 (*Ins*OE1475F)), the OE1475F ORF is split into two pieces, separated by plasmid sequence (Fig. 4B). The correct construction was confirmed by PCR (Fig. 4C) as well as Southern blot (Fig. S1B in File S1).

**Figure 5:**
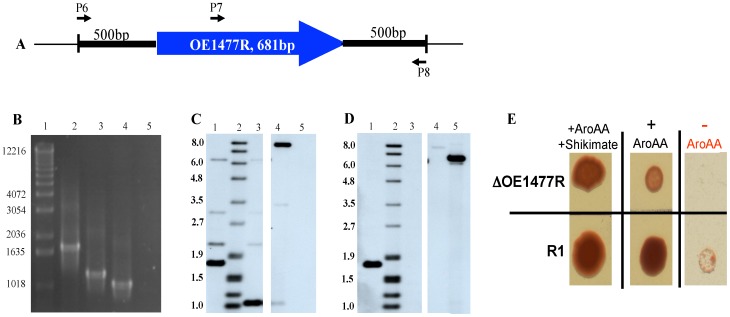
*In vivo* analysis of *H. salinarum* mutant ΔOE1477R. **A**, Illustration of the relative positions of the primers (P6, P7, P8) used in the PCR analysis. **B**, 1% agarose gel of chromosomal DNA from WT and ΔOE1477R using primers P6+P8 (lanes 2 and 4, respectively) and primers P7+P8 (lanes 3 and 5, respectively). MW markers are shown in lane 1. **C** and **D**, Southern blot analysis of WT and ΔOE1477R. Lane 1: ***SacI*** digested DNA from WT, lane 2: DIG VII size marker (in bp), lane 3: *SacI* digested DNA from ΔOE1477R, lane 4: *HindIII* digested pMG700 (containing flanking regions of OE1477R), and lane 5: *HindIII* digested pMG750 (containing the gene OE1477R). C, Hybridization with a DIG labeled probe of the US flanking region of OE1477R. D, Hybridization with a DIG labeled probe of part of the gene. Controls included DNA from plasmids pMG700 and pMG750, respectively, to demonstrate the substrate specificity. **E**, Phenotype of OE1477R compared to WT, growing on different plates. Cells were grown in liquid synthetic medium supplemented with 1.1 mM AroAA and 1.1 mM shikimate until OD_600nm_ = 1.56, and 1.31 (deletion strain and WT, respectively). Cells were washed with basal salt solution before diluting 1/1000 and spotting 3 µl from each dilution on plates supplemented with 1.1 mM AroAA and 1.1 mM shikimate. The viability of the cells and the ability of the deletion strain to grow on these plates are demonstrated in the left and middle panels. While the growth of WT cells was clearly reduced but visible without AroAA (lower right panel), ΔOE1477R required the addition of AroAA.

For the remaining ORF, OE1477R, an in-frame deletion was obtained by integration of a plasmid carrying only the flanking regions of the gene. Confirmation of the correct construct was obtained by PCRs (Fig. 5B). The PCR-confirmed strain was further analyzed by Southern blot hybridization (Fig. 5 C–D), which revealed that it is indeed the desired deletion strain. Growth on agar plates confirmed the genotype (Fig. 5D): wild type cells showed little but clearly visible growth even without the addition of AroAA, while the deletion strain showed reduced growth in the presence of AroAA's and no growth at all in their absence.

### Nutrient requirements of the mutants

**Figure 6:**
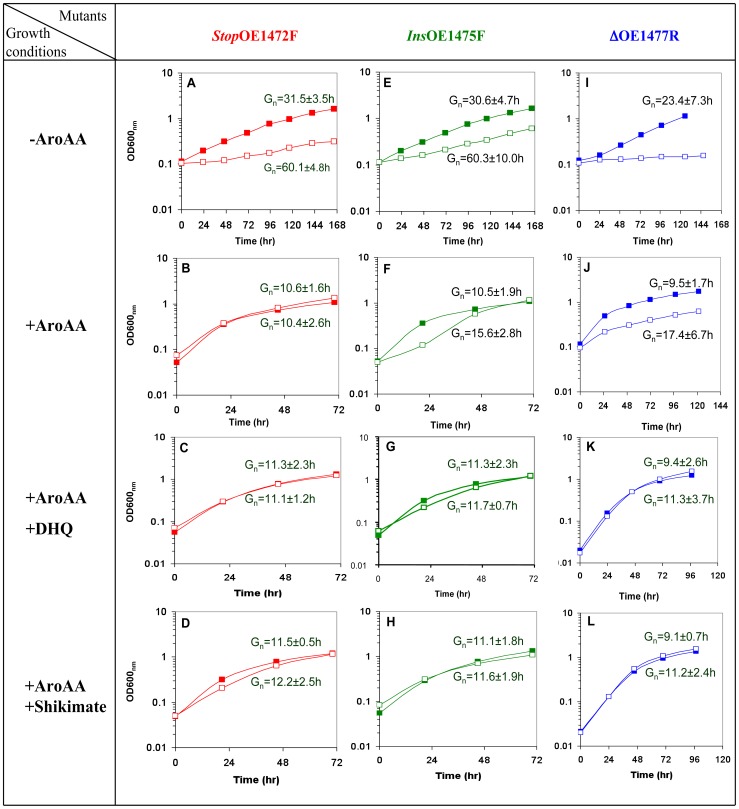
Representative growth curves of mutants *Stop*OE1472F, *Ins*OE1475F and ΔOE1477R. The WT (**▪**) and mutants cells (**□**) grown in synthetic medium with different supplements. Without AroAA (**A**, **E**, and **I**), with 1.1 mM AroAA (**B**, **F**, and **J**), with 1.1 mM AroAA and 1.1 mM DHQ (**C**, **G**, and **K**), and with 1.1 mM AroAA and 1.1 mM shikimate (**D**, **H** and **L**). Inocula were washed with basal salt solution before resuspending in the appropriate medium. Gn times shown above the lines are for WT cells, and Gn times below the lines are for mutants. The cells were grown in 25 ml flasks containing 10 ml medium, at 39°C, and shaken at 100 rpm.

To quantitatively assess the roles of the proposed ORFs in AroAA biosynthesis, cell growth was measured in synthetic media containing AroAA and or precursors, i.e. (1) no AroAA, (2) 1.1 mM AroAA, (3) 1.1 mM AroAA+1.1 mM DHQ, or (4) 1.1 mM AroAA+ 1.1 mM shikimate (Fig. 6).

The mutants, *Stop*OE1472F, and *Ins*OE1475F showed a 2-fold reduction in growth rate in media without AroAA (Fig. 6A, and 6E, respectively). Addition of AroAA allowed mutant *Stop*OE1472F to grow to the same level as WT (Fig. 6B), while mutant *Ins*OE1475F required additional supplementation (Fig. 6F). The deletion strain, ΔOE1477R, was incapable of growing without AroAA in the medium (Fig. 6I), but could grow at half the rate of the WT when supplemented with AroAA (Fig. 6J). The ability of AroAA to compensate for the loss of the enzymes encoded by ORFs OE1472F, OE1475F and OE1477R indicates they are likely to be involved in the AroAA biosynthesis, supporting the hypothesis that *H*. *salinarum* uses a pathway similar to that of *M*. *jannaschii*.

As expected, the deletion mutant ΔOE1477R was auxotrophic for AroAA, with exogenous supplementation restoring about half of the WT growth rate. Surprisingly, further supplementation with DHQ allowed the cells to reach WT levels (Fig. 6K), suggesting that the gene product of OE1477R acts on a substrate upstream to DHQ, which would be inconsistent with the proposed pathway (Fig. 1B). However, LC-MS analyses (table S1 in file S1) showed that 3% dehydroshikimate (DHS) forms spontaneously during incubation of DHQ at 37°C in a salt solution (without cells). Consequently, cells incubated with only DHQ also received at least 0.033 mM DHS, explaining the growth promoting effect of DHQ addition. These results are consistent with the assignment of ORF OE1477R as gene *aro*D (Fig. 1). Below it is shown that its gene product indeed has DHQ dehydratase activity.

### The mutants act on substrates upstream to Shikimate

As demonstrated above, the enzymes encoded by ORFs OE1472F, OE1475F and OE1477R (*aro*D) are involved in the AroAA biosynthesis pathway. Furthermore, the mutants *Ins*OE1475F and ΔOE1477R required supplementation of AroAA plus either DHQ or shikimate (Fig. 6G–H and K–L, respectively). Therefore it was of interest to check what would be the influence of either DHQ or shikimate alone on the mutants.

**Figure 7:**
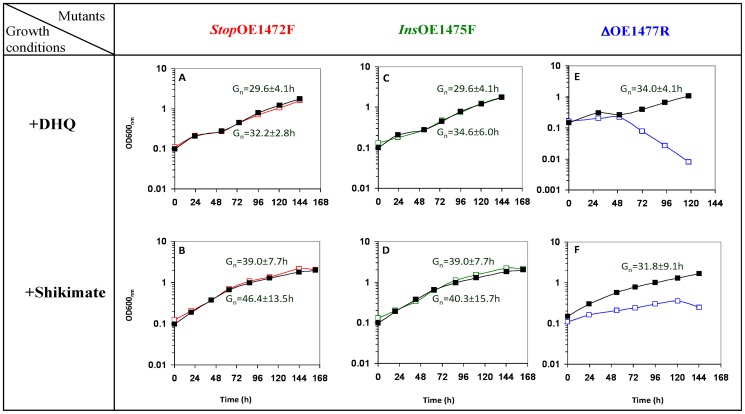
Representative growth curves of mutants *Stop*OE1472F, *Ins*OE1475F and ΔOE1477R in synthetic medium supplemented with either 1.1 mM DHQ (A, C and E) or 1.1 mM Shikimate (B, D and F). WT (**▪**) and mutant cells (**□**) were grown as described for Fig. 6. Gn times shown above the lines are for WT cells, and Gn times below the lines are for mutants. The growth of mutant ΔOE1477R in synthetic medium supplemented with DHQ was linear.(Fig.7F).

As seen in Fig. 7A–D, mutants *Stop*OE1472F and *Ins*OE1475F could grow to the same level as WT when grown in media with only DHQ or shikimate, indicating that the enzymes corresponding to these ORFs act upstream to both DHQ and shikimate. The average Gn times obtained in these media are similar to that obtained when growing the WT cells in synthetic medium without AroAA (Fig. 6A). Addition of AroAA plus DHQ or shikimate improved the growth rate of the mutants *Stop*OE1472F and *Ins*OE1475F (Fig. 6C–D, G–H, respectively).

As expected, the deletion strain ΔOE1477R was unable to grow in medium containing only DHQ. The spontaneous formation of DHS was not sufficient to support growth, leading to cell death after 48 h (Fig. 7E). However, the deletion strain could grow to some extent (but only linearly) in medium supplemented with only shikimate, suggesting that the enzyme encoded by ORF OE1477R acts on a substrate upstream to shikimate (Fig. 7F).

### DHQ and Shikimate induce the uptake of Phe in mutants InsOE1475F and ΔOE1477R

The enhanced growth of mutants when media were supplemented with AroAA, AroAA plus DHQ, or AroAA plus shikimate, indicated that the mutants were able to take up AroAA and use them for growth. To establish that uptake was occurring, the extracellular concentrations of phenylalanine (Phe) and tyrosine (Tyr) were measured during the growth. This allowed calculation of consumption rates of these AroAA. The uptake of Tryptophan (Trp) could not be measured in this the assay.

Media were inoculated with either WT or mutant strains and the levels of Phe and Tyr in the media measured at different time points during growth. The decrease in extracellular concentrations of Phe and Tyr during growth (consumption rates) were modeled as follows: the rate at a given time was defined to be proportional to the cellular density, and model parameters were obtained by minimization of the residual error (see materials and methods).

**Figure 8:**
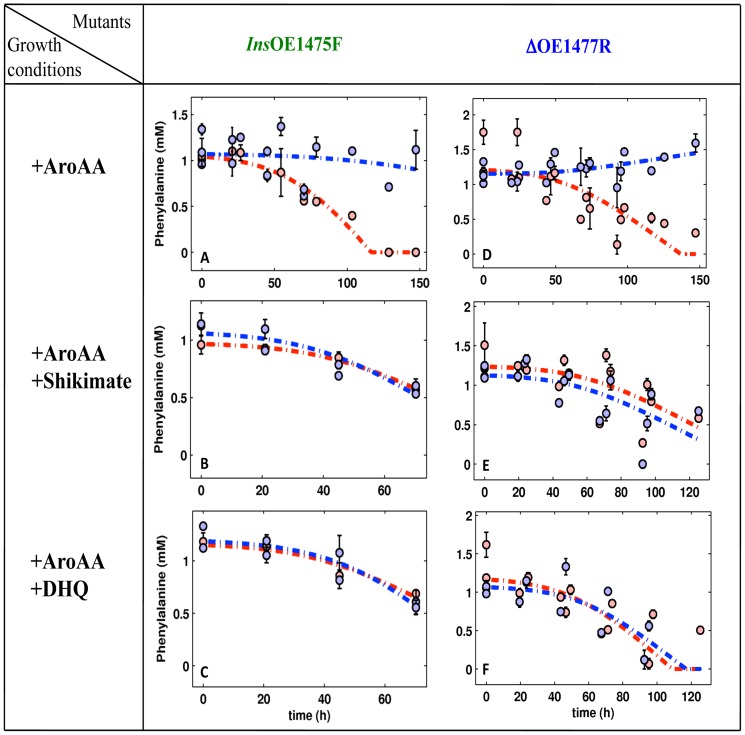
Phe consumption during the growth of WT and mutants *Ins*OE1475F (left panel) ΔOE1477R (right panel). Culture media were supplemented with AroAAs, DHQ or shikimate, as indicated in the growth conditions column at left (see Methods for details). In each graph, the red circles represent Phe consumption by WT *H. salinarum* and blue circles represent consumption by the mutant strain. Simulation curves (dashed lines), are red for WT, and blue for the mutants. Error bars show the deviation of the average calculated from three biological repeats and two technical repeats. The uptake rates were calculated from the corresponding simulations.

Tyr levels did not change significantly in all mutants (data not shown), but Phe levels dropped substantially, allowing uptake rates to be determined (Fig. 8 and table 1). WT cells took up all the Phe supplied in the medium, as did the *Stop*OE1472F mutant. Mutants *Ins*OE1475F and ΔOE1477R were unable to take up Phe (Fig. 8A, 8D) unless the AroAA were supplemented with either DHQ or shikimate, which stimulated Phe uptake to that of the WT (Fig. 8B–C, E–F and table 1).

**Table 1:** Uptake of phenylalanine by WT and mutant strains of *H. salinarum* grown in synthetic media with different supplementations.

Synthetic medium supplemented with	Uptake of Phe by *H. salinarum* strains(1)			
	WT	*Stop*OE1472F	*Ins*OE1475F	ΔOE1477R
+AroAA	11.9±0.8	11.2±1.4	0	1.3
+AroAA+DHQ	12.4±1.4	11.9±1.5	16.7±0.5	10.7±1.9
+AroAA+Shikimate	10.7±1.4	10.6±1.5	12.4±1.62	10.8±2.2

(1) Uptake rates are nmole OD^−1^ml^−1^h^−1^, and were calculated from the corresponding model simulations. For more information see materials and methods and Gonzalez et al., [5].

### Activity of the purified gene products OE1472F and OE1477R

In order to study the functional properties of the protein products of ORFs OE1472F and OE1477R, these genes were overexpressed in *E*. *coli*, the resulting proteins purified, and their enzymic properties examined (see materials and methods).

OE1472F (homolog to MJ0400) was predicted to catalyze the transaldolase reaction between DKFP and ASA, while OE2019F (homolog to MJ1585) was predicted to be a F-1,6-P aldolase (EC 4.1.2.13). Unexpectedly, the proteins of both OE1472F and OE2019F displayed similar F-1,6-P aldolase activity, as measured by the colorimetric assay (Table 2, *H*. *salinarum*).

**Table 2:** Aldolase and transaldolase activities reported in Archaea.

Organism	Assay	Specific activity (mU mg Protein^−1^)			Reference
		ORF	Substrate	activity	
*M*. *jannaschii*	(1)coupled assay, recombinant proteins	(4) MJ0400 (5) MJ1585	F-1,6-P	<0.1 540	[25]
*M*. *maripaludis*	(1) coupled assay, cell extract	WT, (4) ΔMMP0686	F-1,6-P	6.6–7.2 6.6–7.2	[25]
*M*. *jannaschii*	(2) GC-MS, recombinant proteins	(4) MJ0400+ (6) MJ1249 MJ0400+MJ1249+NADP MJ0400+MJ1249+NAD	ASA+DKFP	4.8 103.1 226.8	[6]
*H*. *salinarum*	(3) colorimetric assay, recombinant proteins	(4) OE1472F (5) OE2019F	F-1,6-P	71 78	This study
(7) *T. tenax*	(1) coupled assay, recombinant proteins	AJ310483(9)	F-1,6-P	230	[10]
(8) *P. furiosus*	(1) coupled assay, recombinant proteins	AF368259(9)	F-1,6-P	580	[10]

(1) In the coupled assay, aldolase activity was determined using coupled assay, were the cleavage of F-1,6-P was coupled with glycerol-3-phosphate dehydrogenase (EC 1.1.1.8) and triose-phosphate isomerase (TIM, EC 5.3.1.1) of rabbit muscle. Enzymatic activities were measured by monitoring the increase in absorption of NADH at 366 nm (ε_50°C_ = 3.36 mm^−1^cm^−1^).

(2) DHQ generated with recombinant proteins was determined by GC-MS.

(3) Formation of DHAP was measured by Colorimetric assay (see materials and methods for details).

(4) **MJ0400** from *M*. *jannaschii* is homologous to OE1472F from *H*. *salinarum* and MMP0686 from *M*. *maripaludis*. Predicted transaldolase catalyzing the first reaction of the AroAA biosynthesis pathway in these organisms.

(5) **MJ1585** from *M*. *jannaschii* is homologous to OE2019F from *H*. *salinarum* and MMP0293 from *M*. *maripaludis*. It is an aldolase.

(6) **MJ1249** is homologous to OE1475F from *H*. *salinarum* and MMP0006 from *M*. *maripaludis*. It is believed to catalyze the second reaction in the AroAA biosynthesis, synthesizing DHQ.

(7) *Thermoproteus tenax*, crenarchaeon.

(8) *Pyrococcus furiosus*, euryarchaeon.

(9) GenBank accession number.

**Figure 9:**
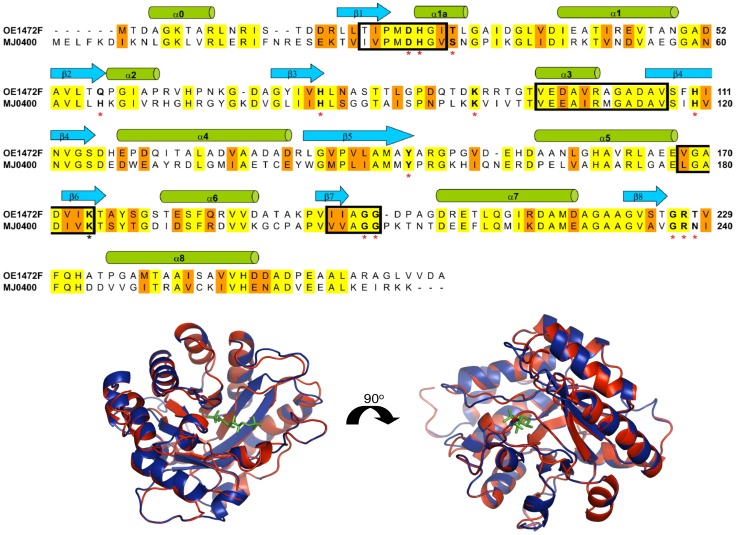
Protein sequence alignment of OE1472F from *H*. *salinarum* and MJ0400 from *M*. *jannaschii*. Conserved sequence motifs of archaea, described by Siebers et al. [10], are marked with black boxes. Active site residues described by Morar et al. for *M*. *jannaschii* [9] are marked in boldface letters and red asterisks. The catalytic lysine residue (Lys237) determined for the *E*. *coli* class IA aldolase (DhnA type aldolase), is marked with a black asterisk [44]. Identical residues are colored yellow (43%), and similar residues are colored orange (61%). The alignment was done using ClustalW multiple sequence alignment program (http://us.expasy.org). The secondary structure elements according to MJ0400 [9] are shown at the top of the alignment. Lower panel: Superposition of MJ0400-F1,6-P complex (PDB:2QJG) with OE1472F. Colours indicate: Blue, MJ0400; red, OE1472F; green, F-1,6-P. The PDB file of OE1472F was generated by Phyre (http://us.expasy.org) and 3D structure was superimposed and viewed by Pymol (http://www.pymol.org/).

Given the demonstrated involvement of OE1472F in AroAA biosynthesis (see earlier sections) and its strong homology to MJ0400 (Fig. 9), we expected this ORF to encode a transaldolase that acts on ASA and DKFP. Although derivatized ASA, DKFP, F-1,6-P, DHAP, GAP and DHQ were detected by LC-MS (materials and methods and retention times, table S2 in file S1), an LC-MS assay showed no transaldolase activity for OE1472F. This was unlikely to be due to the high salt interfering with detection, as the substrates in this assay were able to be derivatized, detected and identified The most likely reason for the lack of activity detected by LC-MS is that the specific activity of the purified protein is below the limit of detection of the assay.

As expected, the purified gene product of ORF OE1477R was shown to have dehydroquinate dehydratase activity, i.e. the formation of DHS from DHQ, so proving its *aro*D function. The specific activity of the purified gene product of OE1477R from *H*. *salinarum* was 55±6 mU mg protein^−1^.

### Transcription of AroAA-related genes

To further examine the AroAA biosynthetic pathway and to identify the functions of relevant genes other than ORFs OE1472F, OE1475F and *aro*D, a genome-wide microarray was used to examine the activities of 3072 predicted ORFs of *H*. *salinarum* [11]. Changes in the transcript levels of selected genes were investigated in more detail by RT-PCR. This was expected to assist in identifying the pathways of AroAA production, as well as shed light on associated regulatory or metabolic genes.

### Genes coding for the biosynthesis pathway

As the presence of AroAA is assumed to suppress the expression of genes involved in AroAA biosynthesis, the transcription of AroAA-related genes was compared between cells grown with and without AroAA (Table S3 in file S1). The comparison was performed at two distinct stages of cell growth (OD_600 nm_ = 0.2 and 0.58). For three genes of the pathway (ORFs OE1472F, OE1475F and OE1477R), transcription was measured both by microarray and RT-PCR. Due to the high sensitivity of the RT-PCR, genes with fold induction (table S3 in file S1) above 4 were designated as strongly induced, while for the microarray, a fold induction above 2 was regarded as strongly induced.

**Figure 10:**
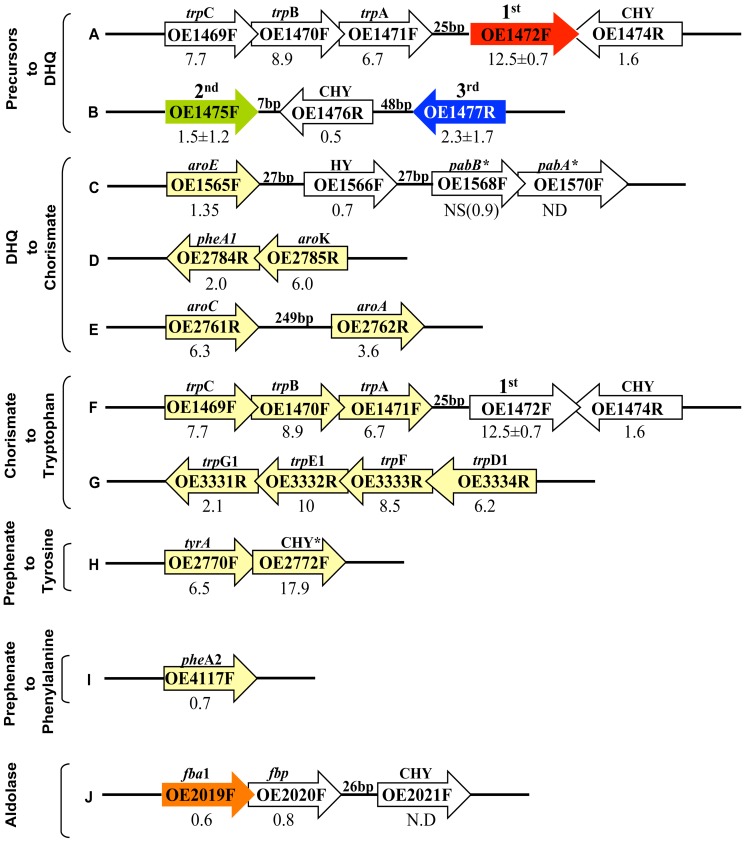
Genomic contexts of ORFs involved AroAA biosynthesis in *H*. *salinarum*. Arrows show the relative positions and orientations of ORFs (but are not drawn to scale); those colored in red, green, blue or yellow represent ORFs assigned to the AroAA biosynthesis pathway while other, nearby ORFs, are uncoloured. **A** and **B**, ORFs assigned to convert precursors to DHQ. The first and second ORFs (OE1472F in red and OE1475F in green) are homologs of *M*. *jannaschii* ORFs [6]. These form part of the non-canonical part of the pathway. ORF OE1477R (in blue) is the 3^rd^ ORF discussed in this paper. **C** and **D**, ORFs needed to convert DHQ to chorismate. **F** and **G**, the tryptophan branch. **H**, the tyrosine branch, **I**-the phenylalanine branch. J- the genomic context of ORF OE2019F, homolog to OE1472F. The numbers below the arrows represent the induction of AroAA-related genes in *H*. *salinarum* R1 cells grown in synthetic medium without AroAA relative to synthetic medium with AroAA. Induction values ≥2.0 are marked in red, and have p values of ≤10^−3^ unless indicated. * The two ORFs are involved in the conversion of chorismate to para-aminobenzoate, an intermediate of folate biosynthesis, **NS**, not significant; **ND**, not detected; **CHY**, conserved hypothetical protein; **HY**, hypothetical protein. ORF names and predicted functions are derived from the genome annotation at www.halolex.mpg.de.

The transcription levels of ORFs OE1472F, OE1475F and OE1477R, as assessed by RT-PCR, are shown in Fig. 10A–B (and table S3 in file S1). The expression of OE1472F was strongly induced (12.5±0.7 fold), while ORF OE1475F remained unchanged, and was similar to OE1477R (1.5±1.2 and 2.3±1.7, respectively). The genes involved in the formation of chorismate from DHQ (Fig. 10C–E) displayed significant levels of transcriptional regulation when assessed by the microarray. Of these genes, *aro*K, *aro*A, and *aro*C gave high values (3.6–6 fold induction) while *aro*D showed no significant change (1.6 fold induction). In both the RT-PCR and microarray assays, ORFs OE1475F and OE1477R displayed similar rates of induction. In the branch leading from chorismate to tryptophan (Fig. 10F–G), transcription of all 7 ORFs was significantly induced during early exponential growth phase, and decreased later in the mid-exponential phase (OD_600 nm_ = 0.58). The parallel changes in gene expression of these ORFs are consistent with their genomic arrangement (*trp*CBA-OE1472F, *trp*D_1_FE_1_G_1_), suggesting they are organized into two distinct operons.

In the proposed pathway for de novo AroAA biosynthesis (Fig. 1A), the first step is catalyzed by the OE1472F protein, which cleaves the six carbon substrate, DKFP, followed by condensation with ASA. According to the genomic data of *H*. *salinarum* (www.halolex.mpg.de), ASA can be synthesized either from homoserine (involving ORF OE4722R) or L-aspartate (involving ORFs OE4333R and OE3063F). The transcription levels of these ORFs showed no change. Moreover, no regulation was observed for ORF OE2500R, which was predicted to synthesize methylglyoxal (precursor for DKFP) from GAP, indicating constitutive induction of this gene.

### Transporters of AroAA

Five transport systems for AroAA are known in *E*. *coli* [12], but in *H*. *salinarum* four of these (*mtr*, *tnaB*, *tyrP* or *pheP*) had no strong homologs. The fifth *E*. *coli* transporter, *aro*P, is known as a general transporter for all amino acids. In *H*. *salinarum*, ORF OE2779F (weak homolog to *aro*P and *phe*P) was predicted to be an amino acid transport protein, and in the current study was strongly induced (3.5 fold) in cells grown without AroAA (Table S3 in file S1), supporting a transport function. Unlike the other genes shown in table S3 in file S1, there was no change in the degree of regulation of this ORF at different growth phases, suggesting that AroAA are still being taken up during mid-exponential phase growth.

Genes of another predicted transporter (*dpp*A_3_B_3_C_3_D_3_F_3_) are arranged in an operon, and were strongly up-regulated (2.9–5.6 fold) in cells grown without AroAA (Fig. S3 and table S3 in file S1). Although the specific substrate of individual ABC transporters is difficult to predict by sequence homology, these results suggest that this transporter is involved in AroAA uptake.

Three ORFs annotated as Na^+^-dependent transporters were also strongly induced when *H*. *salinarum* cells were grown without AroAA (Table S3 in file S1). Na^+^-dependent transporters have been shown to be able to transport amino acids using the Na^+^ gradient as a motive force ([13] and references within).

### Conserved hypothetical proteins and hypothetical proteins

The microarray analysis detected seven ORFs annotated as conserved (CHY) or hypothetical (HY) proteins, which were significantly up regulated in the absence of AroAA in the growth medium. While the data indicated that these ORFs are important in the cellular response to AroAA limitation, homology searches did not reveal any conserved domains within the predicted protein sequences that would give a clue to their function. It was also found that some members of this group lie in close proximity to each other, and are likely to be co-transcribed, i.e. form operons (Fig. S3 and table S3 in file S1).

### Transcription factors and regulators

Transcription in archaea is catalyzed by a eukaryotic type RNA polymerase that requires only two transcription factors, TATA-binding protein (TBP) and transcription factor B (TFB). Multiple members of these two transcription factors have been identified in *H*. *salinarum* (six TBPs, and seven TFBs) [14], [15], but in the present study only TBPe (ORF OE4146F) was strongly up regulated (3.2 fold) by addition of AroAA to the growth medium. Transcription of the TFBs remained unchanged. These results are consistent with those of Facciotti *et al.* [15], who suggested a dominant role for TBPe.

*H*. *salinarum* possesses seven Lrp-like transcriptional regulators (*trh*1 to *trh*7), which have homologues that are widely distributed across bacterial and archaeal species [16]. Their specific role in the regulation of *H*. *salinarum* is not well known except for the information from two studies by Bonneau *et al*., and Schwaiger *et al*. [17], [18]. The DNA microarray results obtained in the current study showed that ORF OE2776F (*trh1* = LrpA2) was strongly induced (14.9 fold) when WT cells were grown without AroAA, suggesting LrpA2 is a regulator of AroAA biosynthesis. There is still a need to determine which ORFs are controlled by this regulator, and whether this control is direct or indirect.

## Discussion

The putative functions of ORFs OE1472F, OE1475F and OE1477R were confirmed experimentally, both *in vivo* and *in vitro*. The first two ORFs were found to be part of a non-canonical pathway for AroAA biosynthesis in *H*. *salinarum*, while ORF OE1477R (*aro*D) is the first ORF in the canonical pathway. All three ORFs act on substrates upstream to shikimate, as demonstrated by the phenotypes of mutant strains defective in these ORFs. The set of reactions leading to the formation of AroAA (i.e. the proposed pathway, based on *M*. *jannaschii*) is represented in Fig. 1.

The first ORF in this pathway, ORF OE1472F, was classified as a member of the archaeal aldolase/transaldolase family based on sequence alignment and its close structural similarity to the transaldolase from *M*. *jannaschii*. The sequence alignment of ORFs OE1472F and MJ0400 (Fig. 9) shows that (1) 43% of the amino acids in OE1472F are completely conserved while 61% of the residues are similar, (2) all the active site residues described by Morar *et al.* for *M*. *jannaschii* [9] can be identified in OE1472F, and all are completely conserved, and (3) all the conserved sequence motifs described by Siebers *et al.* [10] in archaea can be identified. Moreover, if the structure of the MJ0400-F1,6-P complex (Fig. 9, lower panel, in blue) is overlayed with the predicted structure for OE1472F (Fig. 9, lower panel, in red), there are almost no differences except in the unstructured loop regions (root mean deviation value of 1.6 for 256 residues out of 258 residues of OE1472F). Although, transaldolase activity towards ASA and DKFP could not be shown experimentally in this study, five lines of evidence suggest that ORF OE1472F is the first gene in the de novo AroAA pathway in *H*. *salinarum*. First, ORF OE1472F is part of the *trp*CBA-OE1472F operon, as shown previously in studies that mapped transcription start and termination sites [19], [20]. In addition, the small, 25 bp gap between ORF OE1471F and ORF OE1472F does not contain any TATA box sequence. Second, transcriptions of all ORFs involved in this operon were strongly induced in cells grown in the absence of AroAA (Fig. 10F). Third, the transcription levels of *trp*CBA and *trp*D_1_FE_1_G*1* were 1.5–5 fold up regulated in mutant *Stop*OE1472F grown in synthetic medium without AroAA (Fig S2 in file S1), suggesting that the reduced transcription levels of OE1472F in mutant *Stop*OE1472F result in cells sensing a greater need for AroAA (than wt cells). Fourth, mutant *Stop*OE1472F could only grow to the same level as WT when provided with AroAA. The absence of AroAA in the synthetic medium strongly reduced the ability of the mutant to grow, and thereby confirmed the role of this ORF in the AroAA biosynthesis pathway. The fifth line of evidence that argues in favor of a transaldolase function for ORF OE1472F is the unexpected aldolase activity of this enzyme towards F-1,6-P. As demonstrated previously by Morar *et al.*, for the *M*. *jannaschii* protein MJ0400 [9], and as seen in Fig. 9, both transaldolases can bind F-1,6-P in the active site. While MJ0400 showed no detectable aldolase activity, OE1472F displayed aldolase activity towards F-1,6-P. This aldolase activity is comparable to the specific activity of the *H*. *salinarum* aldolase, OE2019F (Table 2).

**Figure 11:**
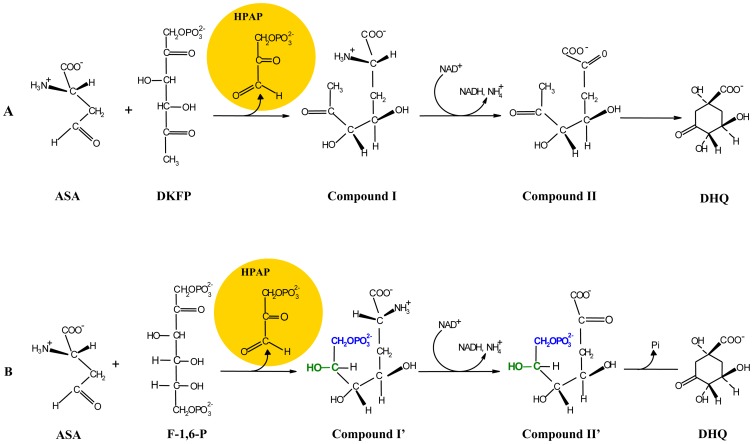
The expected products and intermediates after cleavage of DKFP (A) or F-1,6-P (B) followed by condensation with ASA. The differences between compound I compare to compound I' are labeled in blue and green, respectively. **ASA**, L-aspartate semialdehyde; **DKFP**, 6-deoxy-5-ketofructose 1-phosphate; **F-1,6-P**, fructose 1,6-bisphosphate; **HPAP**, hydroxpyruvaldehyde phosphate; **DHQ**, 3 dehydroquinate.

The unexpected aldolase activity of protein OE1472F suggests two possibilities either (1) ASA and F-1,6-P are the precursors in the AroAA biosynthesis pathway in *H*. *salinarum* (Fig 11), or (2) ORF OE1472F has an additional role in the AroAA biosynthesis pathway, such as the formation of DKFP. The possibilities are discussed in more detail below.

A transaldolase reaction with either DKFP or F-1,6-P (Fig. 11A and B, respectively) will result in the formation of DHQ. HPAP (hydroxpyruvaldehyde phosphate), can be produced from both of these precursors, and as the reaction mechanism of enzyme MJ0400 is likely to be identical to that of OE1472F, it should result in abstraction of a proton from C3 of either DKFP or F-1,6-F. However, two different intermediates will be released for nucleophilic attack on ASA: compound I with DKFP and compound I' with F-1-6-P. Compound I and I' differ by their end groups: I: O = CR-CH_3_ and I':HO-CHR-CH_2_-O-PO_3_^2−^, respectively. In MJ0400, Asp33 acts as a proton acceptor and Lys184 acts as a Schiff base. As both of these amino acids are conserved in OE1472F, they could provide the same functions (Fig. 9). After transamination the same “intermediate” is reached from both I and I' by enolization of I or dephosphorylation of I', namely HO-CR = CH_2_. Finally, nucleophilic attack of the  = CH_2_ group will lead to the formation of DHQ when a transaldolase reaction take place with either DKFP or F-1,6-P. Therefore, one cannot exclude F-1,6-P as a substrate of the AroAA biosynthesis pathway of *H*. *salinarum*. Moreover, the binding of F-1,6-P in the binding site of MJ0400 protein, the structural similarity between MJ0400 and OE1472F as well as the demonstrated aldolase activity of the OE1472F protein from *H*. *salinarum* (Table 2), further support the possibility of ASA and F-1,6-P been the precursors of the AroAA biosynthesis pathway in *H*. *salinarum* (Fig 11B).

**Figure 12:**
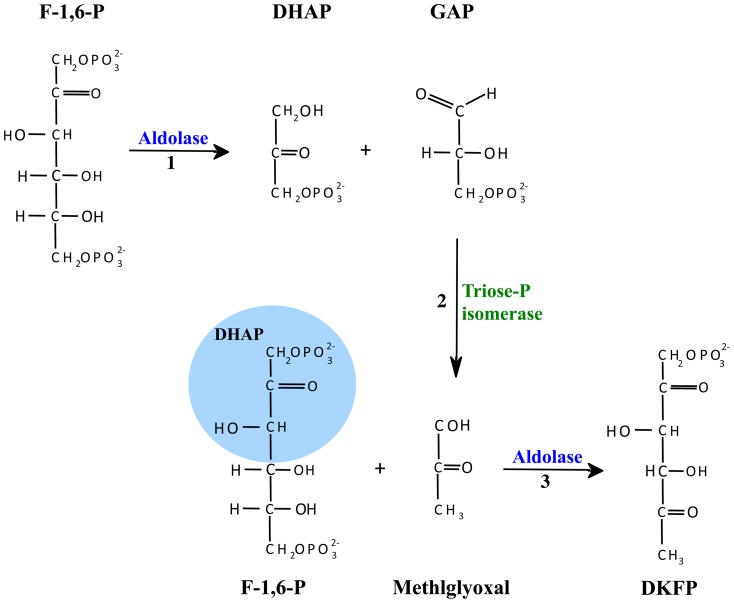
Proposed pathways for the formation of methylglyoxal and DKFP. In *M*. *jannaschii*, reactions 1 and 3 were suggested to be catalyzed by the multifunctional glycolytic enzyme MJ1585. Reaction 2 was suggested to be catalyzed by triose phosphate isomerase EC 5.3.1.1 (ORFs MJ1528 and OE2500R of *M*. *jannaschii* and *H*. *salinarum*, respectively). Modified from White and Xu [24].

It might also be that the aldolase activity demonstrated by ORF OE1472F was due to its involvement in the formation of DKFP. Production of DKFP could be derived from methylglyoxal synthase (EC 4.2.3.3), but while homologs of methylglyoxal synthase have been identified in some sequenced haloarchaeal genomes (such as *Natrinema* sp. J7-2 [22] and *Haloquadratum walsbyi* (accession YP_657298)), no homolog was found in *H*. *salinarum*. Alternatively, DHAP and methylglyoxal may be the precursors of DKFP. DHAP may derive from F-1,6-P whereas methylglyoxal may arise enzymatically from GAP by triose phosphate isomerase (OE2500R (EC 5.3.1.1), Fig. 12 reaction #2) [23]. It was demonstrated by White and Xu [24] that In *M*. *jannaschii* DKFP is generated by condensation of methylglyoxal with a DHAP fragment (derived from F-1-P and/or F-1,6-P). This reaction was shown to be carried out by the product of gene MJ1585 (homolog to OE2019F) (see table 2, experiment 5 and 6 in [24]), leading the authors to conclude that the glycolytic enzyme MJ1585 is a multifunctional enzyme, responsible for both cleavage of F-1,6-P ([25] and Fig. 12 reaction #1) and the formation of DKFP (Fig. 12, reaction #3). However, given the demonstrated aldolase activities of OE2019F and OE1472F, one may speculate that in *H*. *salinarum* the two aldolase reactions depicted in Fig.12 can be catalyzed by either OE2919F and OE1472F (reaction #1 and #3, respectively), or only by OE1472F (reaction #1 and #3). Indirect evidence supporting the possibility that ORF OE1472F can catalyze reaction #3 was given by Morar *et al.* [9]. There, the crystal structures of MJ0400 (PDB:2QJH, homolog to OE1472F) contained a clear DHAP electron density linked to Lys184 located on strand ß6, shown to be one of the active site residues.

Although ORF OE1472F demonstrated aldolase activity its role in AroAA biosynthesis pathway was confirmed by the reduced ability of mutant *Stop*OE1472F to grow in the absence of AroAA. Since this mutant can grow to some extent in a medium lacking AroAA, it suggests the following alternatives: 1) the transcription of ORF OE1472F was not completely blocked by the integrated terminator, 2) another gene in *H*. *salinarum* (possibly ORF OE2019F) can complement, to a limited extent, the loss of ORF OE1472F, or 3) a second pathway for the synthesis of AroAA is present in *H*. *salinarum*. The first and second alternatives cannot be excluded. As for the third alternative, although Porat *et al*. have shown that aryl acids are substrates of a second pathway for AroAA biosynthesis in *M*. *maripaludis* [21], it is unlikely in halophilic archaea. The occurrence of AroAA synthesis via indolepyruvate oxidoreductase is unlikely since only two ferredoxin- dependent oxidoreductase complexes have been identified within the halophilic archaea genomes (pyruvate- and 2-oxoglutarate-ferredoxin oxidoreductase, OE2623R, OE2622R, and OE1711R, OE1710R, respectively). No homologs of indolepyruvate- ferredoxin oxidoreductase (*iro*A2, EC 1.2.7.8, MMP0713, and MMP0714, the α and the β subunits, respectively) have been found in *H*. *salinarum*. Therefore, if aryl acids can be used by *H*. *salinarum* for AroAA biosynthesis, then the enzymes are unrelated to those of *M*. *maripaludis*.

The second ORF in the AroAA pathway, ORF OE1475F, is believed to be a 3-dehydroquinate synthase, and as such, responsible for the oxidative deamination, phosphorlyation and the cyclization reactions, to form DHQ. In contrast to *H*. *salinarum* and *M*. *jannaschii* (MJ1249), the homolog from *M*. *maripaludis* (ORF MMP0006) has been shown not to be involved in AroAA biosynthesis. Deletion of this gene did not result in a growth requirement for AroAA in *M*. *maripaludis*, and the mutant grew as well as the WT strain (Fig. 5B in [25]). In addition, labeling experiments showed that shikimate and DHS pools were labeled to nearly the same extent as in the WT. Therefore, Porat *et al.* [25] concluded that the gene MMP0006 was not required for AroAA biosynthesis in *M*. *maripaludis*, although it is 69% identical to the *M*. *jannaschii* homolog (Fig. 3 in [25]). On the contrary, in this study *H*. *salinarum* ORF OE1475F was found to play a role in the biosynthesis of AroAA, as the mutant *Ins*OE1475F required AroAA for growth.

The growth impairment of mutant *Ins*OE1475F was restored when cultured in synthetic medium supplemented with all three AroAA. The uptake of Phe was sufficient to support the growth of the mutant. As shown by Gonzalez *et al.* [5], when amino acids are supplied to WT *H*. *salinarum*, in most cases the uptake rates exceed the rate at which they are incorporated into cellular proteins. More specifically, it was proven experimentally that only 0.11 mM Phe OD^−1^ cell biomass was incorporated into biomass, allowing growth to an optical density of 1.8 OD_600 nm_. In the case of mutant *Ins*OE1475F, 0.094 mM Phe OD^−1^ was consumed by the cells when grown in synthetic medium supplemented only by AroAA. This level of Phe consumption was sufficient to support the growth of mutant *Ins*OE1475F.

As demonstrated for mutants *Ins*OE1475F and ΔOE1477R (Table 1), the addition of either DHQ or shikimate to AroAA, stimulates the uptake of Phe from the medium. This suggests that while ORF OE1475F and OE1477R might regulate the specific transporters of AroAA, the gene product of OE1472F has little or no regulatory effect on Phe transport. Consequently, mutant *Stop*OE1472F required only AroAA in order to grow as well as WT, while mutants *Ins*OE1475F and ΔOE1477R required additional supplements (i.e DHQ and shikimate). The positive effect on growth of these intermediates suggests they, or their later intermediates in the pathway, are used to synthesise one or more metabolites other than the supplied AroAA. Even in the classical AroAA pathway, intermediates such as chorismate (Fig. 1) lead to important biomolecules other than AroAA. Chorismate is used to make menaquinone (MQ), a member of the respiratory chain, and is also a starting point for the synthesis of tetrahydrofolate (THF), a cofactor for several enzymes. In the current study, the unsupplemented synthetic medium included folic acid (as a source of THF), but not MQ. Since aerobically grown mutant cells supplied with AroAAs were able to grow without added MQ, one can speculate that MQ may be provided in part by some other metabolic reaction. The growth requirements of mutants *Ins*OE1475F and ΔOE1477R suggest that DHQ or shikimate can be channelled into synthesis of currently unsuspected (but growth promoting) metabolites, and/or MQ.

Finally, evidence was provided to show that the gene product of OE1477R catalyses the third step in the biosynthesis pathway of AroAA in *H*. *salinarum*. The enzyme was classified as 3-dehydroquinate dehydratase (*aro*D), and the purified gene product was capable of forming DHS from DHQ (55±6 mU mg protein^−1^). In comparison, the specific activity of dehydroquinate dehydratase from *E*. *coli* was similar (89 mU mg protein^−1^) [26], whereas the specific activity found in cell extracts of *M*. *maripaludis* was much lower (6.0±0.2 mU mg protein^−1^) [21].

The deletion mutant ΔOE1477R could not grow in medium without AroAA, thereby confirming the role of this gene in AroAA biosynthesis. This mutant grew well on synthetic medium with AroAA and either DHQ or shikimate, suggesting the following possibilities: (1) there is an additional branch point in the pathway other than chorismate, or (2) the synthesis of DHQ can originate from quinate. An additional branch point in the pathway, has been shown previously by Porat *et al*., where 4-aminobenzoate is derived from DHQ and not from chorismate [25]. One cannot exclude this scenario for *H*. *salinarum*, as all synthetic media used in this study contained folic acid, therefore the mutants would not need the precursor, 4-aminobenzoate, in order to synthesize tetrahydrofolate (THF). Alternatively, it was demonstrated for *Gluconobacter* and *Acetomonas oxydans* that quinate is metabolized via DHQ and DHS [27], [28]. Quinate is oxidized to DHQ using a NADP-independent quinate dehydrogenase (EC 1.1.99.25). Although no strong homolog was found in *H*. *salinarum* (possible candidate OE6278R, 25% amino acid identity), one cannot exclude this possibility.

In summary, Archaea show considerable and surprising diversity in metabolic pathways that are otherwise highly conserved in Bacteria and Eukarya, the so called canonical pathways. The AroAA pathway examined here is a good example, as it was not possible to predict what the enzymes or reactions might be before White [6] elucidated the initial biochemical steps in methanogenic Archaea. The challenge remains, as the many recently available genome sequences of Archaea commonly show numerous gaps when attempting to reconstruct their biosynthetic pathways, either because they use the same catalytic steps but with non-homologous enzymes, or because they use novel biochemistry to achieve the same result. It is then necessary to determine the unknown steps experimentally. The evolutionary clues provided by non-canonical enzymes, and the altered metabolic potential resulting from novel biosynthetic steps, are likely to take longer to fully understand but will no doubt lead to deeper insights.

## Materials and Methods

### Materials

The following materials were synthesized by Mr. Jürgen Musiol from the core facility of Max Planck institute of Biochemistry, Germany: L-Aspartate Semialdehyde (ASA) was synthesized according to [29], [30], 6-deoxy-5-ketofructose 1-phosphate (DKFP) according to [6], [31], and 3-dehydroquinate (DHQ) was synthesized according to [32]. Some of the DHQ used in this study was a gift from Prof. Osao Adachi, Department of Biological Chemistry, faculty of Agriculture,Yamaguchi University, Japan.

### Strains and medium

*Halobacterium salinarum R1* (DSM 671) and mutants *Stop*OE1472F (OE1471F:: Terminator), *Ins*OE1475F (OE1475F::pMG501) and ΔOE1477R cells (Table S4 in file S1) were grown in chemically-defined medium, modified from that described by Oesterhelt and Krippahl [33]. The composition of the medium is described in Table S5 in file S1.

Cultures were grown at 39°C, 100 rpm in 25 ml flasks containing 10 ml medium. Where mentioned, synthetic medium was supplemented with either 1.1 mM DHQ or shikimate, designated by AroAA+DHQ or AroAA+shikimate, respectively. Synthetic medium without AroAA did not contain the aromatic amino acids: L-Tyr, L-Phe and L-Trp. Both WT and mutants were transferred three times into the respective media before growth measurements were performed. The initial OD at 600 nm was 0.1 OD. The generation times (Gn) represent the average growing rate from at least 3 independent growth curves. Cell growth was monitored by measuring optical density at 600 nm (OD_600_). The medium of the insertion mutants *Stop*OE1472F and *Ins*OE1475F contained as well 10 µg ml^−^1 Mevinolin (Mev).

### Transformation of *H. salinarum*

Transformation of *H. salinarum* was performed according to the PEG method [34], [35] with minor modifications. 1.5 ml of culture (OD_600_ 0.6–0.8) was centrifuged (5 min 16000 g, RT) and supernatant discarded. Residual medium was removed after additional centrifugation (1 min, 16000 g, RT) using a pipette. The cell pellet was then gently resuspended in 150 µl spheroplasting solution (SPH). To produce spheroplasts, 30 µl of 0.25 M EDTA (pH 8.0 in SPH solution) was added and the mixture was incubated for 5 min at RT. Then 10 µl of DNA (in SPH solution, ∼1 µg DNA) was added and the mixture incubated at RT for 5 min. 190 µl of 60%PEG_600_ (in SPH solution), was added to the tube, followed by rapid mixing of the contents to prevent lysis of the cells due to high local concentration of PEG_600_. After 20 min incubation at RT, 1 ml of Halo medium+15% sucrose was added to the tube in order to dilute the PEG. The mixture was centrifuged for 2 min, 16000 g at RT, and the supernatant discarded. After an additional centrifugation (1 min, 16000 g, RT), residual medium was removed by a micropipette. Finally, the pellet was resuspended gently with 1 ml of Halo medium+15% sucrose and incubated at 37°C, 250 rpm for 12–16 h to allow the cells to restore their S-layer.

All the plasmids in this study (Table S6 in file S1) contained the reporter gene *Bga*H (coding for the halophilic galactosidase *Bga*H) from *Haloferax lucentense* [36], [37]. Therefore, before spreading the transformation mixture, the plates which contained 10 µg ml^−1^ Mev, were smeared with X-gal (150 µl of 20 mg ml^−1^, followed by 30 min incubation under the hood). After cell spreading, the plates were incubated at 37°C in a closed transparent box with ∼10 ml water to prevent the plates from drying out. Usually, transformants became visible after 10–14 days.

### Construction of Mutants

#### *Stop*OE1472F

500 bp of the 3′end of OE1471F were amplified by PCR and ligated to a 55 bp long sequence containing the terminator of the *fla*A operon. The resulting fragment was amplified by PCR using LA Taq polymerase (TaKaRa Bio Inc, Japan), purified from preparative 1% agarose gel, cloned into pCR-2.1 TOPO vector (Invitrogen) and sequenced to confirm that no errors had been introduced. It was than subcloned into pKK100 [38], generating plasmid pMG501 (Fig 3A and Table S6 in file S1), which contained Amp^R^, Mev^R^ markers and *Bga*H as a reporter gene. pMG501 was introduced into strain R1, and transformants were recovered on complex medium plates with Mev and Xgal. Colonies were screened by PCR to confirm the in-frame integration of the terminator using the primers indicated in Fig. 3B.

#### *Ins*OE1475F

The central part of OE1475F was amplified by PCR (540 bp), sequenced and cloned into pKK100 [38], to obtain the integration vector pMG601 (Fig. 4A and Table S6 in file S1). pMG601 contain Amp^R^, Mev^R^ markers and the reporter gene *Bga*H for red/blue screening. Successful integration of pMG601 into the R1 chromosome would slice ORF OE1475F into two pieces, separated by the plasmid sequence (Fig. 4B). A 1.8 Kbp PCR amplimer is expected after integration of plasmid pMG6o1 into R1 chromosome (Fig. 4C).

#### ΔOE1477R

A single, in-frame deletion of ORF OE1477R was achieved by homologous recombination using “suicide plasmids” carrying the flanking regions of the gene, as described by Koch and Oesterhelt [38]. In short, flanking regions with specific restriction sites were amplified, sequenced, and purified from a 1% preparative agarose gel. The cleaved flanking regions were ligated to each other (using T4 DNA ligase). The overnight ligation was followed by PCR using the ligated flanking regions as template. The ligated flanking regions (1031 bp) was cloned into the plasmid pMKK100 [38] using the restriction sites *BamHI* and *HindIII*.

The plasmid used for in-frame deletions of ORF OE1477R (pMG700 Table S6 in file S1) contained the flanking regions of OE1477R along with Amp^R^, and Mev^R^ selection markers, and the *Bga*H gene as the reporter gene for blue/red screening.

The plasmid pMG700, was introduced into *H*. *salinarum* R1 cells using the PEG method [34], [35], and plated on complex medium containing Mev (10 µg ml^−1^) and X-gal. Plasmid integrants (via a single cross-over event) would produce mevinolin resistant transformants that displayed a blue phenotype on agar plates containing X-gal.

Blue colored transformants were then transferred into medium without Mev, which allowed a second cross-over event to take place, excising the plasmid together with its Mevinolin-resistance and ß-galactosidase genes from the genome. On plates containing X-gal without Mevinolin, cells which had undergone a second cross-over were identified based on their red color (due to bacteriorhodopsin and bacterioruberin) in contrast to blue colonies that still contained the plasmid.

While two successive cross-over events between the same homologous regions (i.e. two upstream (US) or two downstream (DS) cross-over events) would lead to restoration of the parental genotype, an US cross-over followed by a DS cross-over (or vice versa) would lead to deletion of the target gene. After the second cross-over event, the target gene bounded by the flanking regions will be eliminated and replaced by the direct fusion of the flanking regions as present on the plasmid. Gene deletions were verified by PCR analysis and Southern blot [39].

### Southern blot analysis

1.5 µg of chromosomal DNA from WT cells and from gene deletion candidates were cleaved with a restriction enzyme, and the DNA fragments separated on a 1% agarose gel. The gel was vacuum-blotted onto a nylon membrane (Hybond-N, Amersham), using essentially the method of Southern [39]. For size determination of the DNA fragments, digoxigenin-labeled DNA molecular marker VIII (Roche diagnostic, Mannheim, Germany) was included on the gel. All gel pretreatment steps, such depurination, denaturation and neutralization were done as recommended by Southern [39]. After UV-crosslinking the DNA to the membrane, all further steps, including prehybridzation, hybridization, washing and chemiluminescent detection with CSPD, were performed according to the manufacturers recommendations (Roche diagnostic, Mannheim, Germany).

Probes for hybridization were generated by incorporation of digoxigenin-labeled dUTP in a PCR, using DIG labeling mix ^Plus^ (Roche). Two 500 bp labeled probes were designed to hybridize to the up-stream region of the gene and to the internal region of the corresponding gene. Labeled probes used for Fig. S1 in File S1 were generated according to GE Healthcare recommendations.

### Expression and purification of ORFs OE1472F, OE1477R and OE2019F

*E*. *coli* DH3-Rosetta (Invitrogen) was used as host strain for the expression plasmids (Table S6 in file S1): OE1472F-His _C-6xhis_/pET22b(+), OE1477R-His _C-6xhis_/pET22b(+), and OE2019F-His _C-6xhis_/pET22b(+), respectively. Pre-cultures were grown at 37°C, 250 rpm and used to inoculate 5 liter flasks containing 1 liter LB + 0.1 mg ml^−1^ ampicillin, giving an initial cell density of 0.1OD_600 nm_. Cells were induced with 1 mM IPTG for 3 h (37°C, 200 rpm) after the OD6_00 nm_ reached ∼ 0.6, then cells were harvested by centrifugation (4000 rpm, 40 min, and 4°C) and the cell pellet from each 2 liter culture was stored at −70°C until further use.

Before purification on a NiNTa affinity column (HisTrp HP, 1 ml from GE Healthcare), the cell pellet was thawed in ice and resuspended with 50 ml binding buffer (50 mM NaH_2_PO_4_+300 mM NaCl) containing DNAase. PMSF (Phenylmethylsulfonyl fluoride) in 2-propanol was added to the suspension to a final concentration of 0.2 mM and the cells were lysed using a French press (2 passages, 1000 psi). After centrifugation at 40000 rpm, 40 min, 4°C (L7-55 Beckmann ultra centrifuge rotor Ti50), the clear supernatant was diluted to a final volume of 250 ml with binding buffer. The NiNTa column was equilibrated with the same buffer, and the bound fraction eluted with a step gradient containing 20–250 mM Imidazole. Proteins were analysed by SDS-PAGE and western blots. The three proteins were over-expressed predominantly as soluble proteins. The N-terminal sequences of each of the purified proteins, and their molecular masses were determined by ESI-MS, confirming that the proteins were His-tagged and in full length.

### Activity assays

**Aldolase activity** was assayed by the colorimetric procedure of Silbley and Lehninger [40] with some modifications. Activity was assayed in a 0.5 ml reaction mixture in the presence of 2M KCl with 78.7 mM Tris HCl (pH = 7.2), 1 mM Cysteine, 0.5 mM FeCl_2_, 56 µM hydrazine sulfate (in 50 mM Tris HCl pH = 7.2) and 1.2 µM F-1,6-P, as substrate. The reaction (37°C, 30 min) was terminated with 2 ml of 10% TCA, and the chromogens developed were read at 540 nm. Blank tubes contained 1.2 µM F-1,6-P, which was added after the addition of TCA. Aldolase activity was expressed as µmoles DHAP formed/mg protein/30 min at 37°C.

**3-dehydroquinate dehydratase activity** was assayed by monitoring the formation of 3-dehydroshikimate (DHS) at 234 nm (ε = 12×10^3^ M^−1^cm^−1^) at RT. The standard assay mixture (1 ml) contained 2.625M KCl+43.75 mM Tris-HCl pH = 7.2 and 10 mM 3-dehydroquinate (DHQ, synthesized by Mr. Jürgen Musiol) [41].

### LC-MS

The enzyme activity of recombinant proteins was measured after derivatization of the reaction products and subsequent analysis by LC-MS. ∼51 µg enzyme in 3M KCl +50 mM Tris HCl pH = 7.2 were mixed with 2.5 mM F-1,6-P or 2.5 mM DKFP and 2.5 mM ASA, in a total volume of 60 µl, and incubated at 37°C for 30 min or overnight. For the derivatization, 2 mM o-(-4-nitrobenzyl)-hydroxylamin-hydrochlorid (NBHA) in MeOH was added, in a final volume of 150 µl. The mixture was centrifuged (5 min, 16000 g) and the supernatant was incubated for 1 h at 60°C.

Up to 10 µl of the derivatized mixture was loaded on a C18 reverse phase column (25°C, flow-250 µl/min). Derivatized reactants and products were eluted with ACN (2 min 10% ACN, 28 min gradient from 10%–30%, 5 min 70% ACN, 2 min 90% ACN, 11 min 10% ACN), and monitoring was at 210 nm and 254 nm.

The activity of commercial Aldolase was measured using LC-MS. 16.7 U ml^−1^ TIM, 1.92 mM F-1,6-P, 14.84 mU ml^−1^ Aldolase in 84 mM Tris-HCl pH = 7.2 in total volume of 60 µl were incubated at 37°C, for 30 min. The derivatization with NBHA, and the elution was done as described above.

### Microarrays

The microarray was designed as described by Twellmeyer *et al*., [11]. Each γ - amino-silane coated CMT-GAPS-II glass slide was spotted with five replicates of 2774 DNA probes, manufactured as described by Twellmeyer *et al*., [11].

#### Isolation of total RNA

9 ml cultures of *H*. *salinarum* R1, grown in different media and to different OD_600 nm_, were harvested by centrifugation (5 min, 8000 rpm, 4°C). The pellet was resuspended with 6 ml of peqGOLD RNApure (peqLAB Biotechnology, Erlangen, Germany), and the mixture was frozen in liquid nitrogen and stored at −80°C. Total RNA was extracted using chloroform/phenol extractions, washing with 75% EtOH and finally the RNA was dissolved in 100 µl DEPC-treated water. The RNA was treated with DNAase (DNA-free, Ambion, Huntington, United Kingdom) and the absence of DNA was confirmed by PCR using the following reagents and conditions: LA Taq, primers P1 and P2 for ORF OE1472F (500 bp), 35 cycles and RNA before and after digestion as templates. No band was amplified in the DNAse treated RNA preparation. The quality of the DNA-free total RNA was assessed by 1% denaturating agarose gel in TBE buffer. The concentration of intact total RNA was determined using NanoDrop ND-1000 spectrophotometer (NanoDrop) and the formula 1OD_260 nm_ = 40 ng ml^−1^ ssRNA.

#### Microarray analysis

Medium-adapted cultures of *H*. *salinarum* were grown in synthetic medium with or without AroAA, and cells harvested by centrifugation after growth had reached OD_600 nm_ = 0.2 and 0.58, respectively. RNA was isolated from cell samples as described above. 3 µg of total RNA was reversed-transcribed into cy5 or Cy3-labeled cDNA using a CyScribe first-strand cDNA synthesis kit with random nonamer primers and Cy5/Cys3-dUTP (Amersham Biosciences, Freiburg, Germany). RNA was removed by alkaline hydrolysis, and the cDNA purified and concentrated as described by Zaigler *et al*., [42]. After prehybridization, labeled cDNA was pipetted onto the microarray which was then sealed in hybridization chamber for overnight at 64°C. The microarrays were washed and dried by centrifugation (5 min, 1500 rpm), as described by [42]. Labeled cDNA from cells grown with AroAA was hybridized with labeled cDNA from cells grown without AroAA (in two different OD's). Hybridized microarray slides were scanned for Cy5 and Cy3 florescence signals using GenePix 4000B scanner (Biozym Seientific GmbH, Hessisch Oldendrof, Germany). Image processing was done with the GenePix Pro 6 (Biozym Scientific GmbH) and the florescence values were processed in the R environment using a program written by G. Welzl [11]. Significant gene regulations were identified by one-sample t-test as implemented in the TIGR MultiExperiment viewer [43]. A gene was considered to be significantly regulated if the regulation factor was two fold or higher, and the calculated P value of the regulated gene was P<0.001. The data obtained from the microarray experiment were deposited at http://www.ebi.ac.uk/miamexpress website under the accession number E-MEXP-3818.

### RT-PCR

1 µg total RNA was reversed transcribed with 400 ng random hexamer primer and 50 U of Reverse-iT RTase Blend (ABgene House, UK). Quantitative PCRs were performed in Bio-Rad iCycler MyiQ single-color Real-time PCR detection system using a SYBR green PCR master mix kit (from AB Applied biosystems), except for the analysis of operons *trp*CBA and *trp*D_1_FE_1_G*1* which was performed in a LightCycler480. The final reaction volume was 25 µl with 0.5 µl of the reverse transcription reaction serving as template. Primers were designed with Primer3 (http://biotools.umassmed.edu/bioapps/primer3_www.cgi) and were used at a final concentration of 0.92 pmole µl^−1^.

Transcript level differences were calculated by a relative quantification approach using an internal standard gene, OE1160R, which encodes ribosomal protein L10.eR. For all calculations, the mean-C_t_ of 2 replicate reactions per primer pair was used. The primer pairs used are specified in table S7 in file S1.

### Amino acid analysis

200 µl samples were taken from cultures of R1 and mutants strains grown in various media, at different time points (∼5 points per growing curve). The samples were centrifuged (5 min, 16000 g, RT), and the supernatant from each time point was divided into two tubes. The supernatants were stored at −20°C until all samples were collected. Amino acids were analyzed using an Amino Acid Analyzer (Biotronik LC3000). The data in Fig. 8 and table 1 represent the analysis for Phe and Tyr from aggregate data of 3–4 cultures that were prepared and processed separately.

### The uptake rate model for amino acid uptake studies

The uptake rate at time t was initially modeled as a function of the OD at time t and the concentration of the metabolite at time t. However, since the second parameter, i.e., the term corresponding to the concentration of the metabolite, was of little value, the uptake rate was modeled just as a function of the current OD. Accordingly:

The constant parameter was determined by solving the inverse problem (i.e., parameter optimization using least-squares optimization), to find the best parameter that minimized the deviation between the model and data. The constant parameter should already be the uptake rate. OD(t), was modeled using a sigmoidal function that was also fitted to the data.
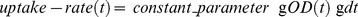



## Supporting Information

File S1 **Supporting files. Figure S1, Southern blot analysis of WT, *stop*EO1472F (A) and *Ins*OE1475F (B).** A, Lane 1: size marker (in bp), lane2: *SacI* digested DNA from WT, lane 3: *SacI* digested DNA from *Stop*OE1472F. B, Lane 1: size marker (in bp), lane2: *NotI* digested DNA from WT, lane 3: *NotI* digested DNA from *Ins*OE1472F. **Figure S2, AroAA regulated CHY and HY ORFs that are closely adjacent, and possibly organized in operons.** Arrows show the relative positions and orientations of ORFs, but are not drawn to scale. The fold regulations are indicated below. *, **, represent ORF overlaps of 10 bp and 3 bp, respectively. The ORFs shown in panels A-D are described in Table 1. The ABC transporter ORFs shown in panel E is described in Table S2 in File S1. **Table S1, Formation of 3-dehydroshikimate (DHS) from 3-dehydroquinate (DHQ) under different conditions. Table S2, Masses detected by LC-MS after derivatization with NBHA. Table S3, Expression of AroAA-related genes and transport-related genes in** ***H*** **.** ***salinarum*** **R1 cells grown in synthetic medium without AroAA relative to synthetic medium with AroAA. Table S4, Strains used in this study. Table S5, The composition of the chemically defined medium, pH = 7.0. Table S6, Plasmids used in this study. Table S7, List of oligonucleotides used in this study.**
(DOCX)
