# Correction: How Do Haloarchaea Synthesize Aromatic Amino Acids?

**DOI:** 10.1371/journal.pone.0116355

**Published:** 2014-12-19

**Authors:** The PLOS ONE Staff

In the Materials and Methods section, there is an error in the equation of the section titled “The uptake rate model for amino acid uptake studies.” Please review the complete, correct equation here: 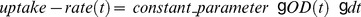
In Table 2, there should not be any commas between the numbers in the ORF and activity columns. Please see the corrected Table 2 here.

**Table 2:** Aldolase and transaldolase activities reported in Archaea.

Organism	Assay	Specific activity (mU mg Protein^-1^)			Reference
		ORF	Substrate	activity	
*M*. *jannaschii*	(1)coupled assay, recombinant proteins	(4) MJ0400, (5) MJ1585	F-1,6-P	<0.1, 540	[25]
*M*. *maripaludis*	(1) coupled assay, cell extract	WT, (4) ΔMMP0686	F-1,6-P	6.6-7.2, 6.6-7.2	[25]
*M*. *jannaschii*	(2) GC-MS, recombinant proteins	(4) MJ0400+ (6) MJ1249, MJ0400+MJ1249+NADP, MJ0400+MJ1249+NAD	ASA+DKFP	4.8, 103.1, 226.8	[6]
*H*. *salinarum*	(3) colorimetric assay, recombinant proteins	(4) OE1472F, (5) OE2019F	F-1,6-P	71, 78	This study
(7) *T. tenax*	(1) coupled assay, recombinant proteins	AJ310483(9)	F-1,6-P	230	[10]
(8) *P. furiosus*	(1) coupled assay, recombinant proteins	AF368259(9)	F-1,6-P	580	[10]

(1) In the coupled assay, aldolase activity was determined using coupled assay, were the cleavage of F-1,6-P was coupled with glycerol-3-phosphate dehydrogenase (EC 1.1.1.8) and triose-phosphate isomerase (TIM, EC 5.3.1.1) of rabbit muscle. Enzymatic activities were measured by monitoring the increase in absorption of NADH at 366 nm (ε_50°C_  =  3.36 mm ^−1^cm^−1^).

(2) DHQ generated with recombinant proteins was determined by GC-MS.

(3) Formation of DHAP was measured by Colorimetric assay (see materials and methods for details).

(4) **MJ0400** from *M*. *jannaschii* is homologous to OE1472F from *H*. *salinarum* and MMP0686 from *M*. *maripaludis*. Predicted transaldolase catalyzing the first reaction of the AroAA biosynthesis pathway in these organisms.

(5) **MJ1585** from *M*. *jannaschii* is homologous to OE2019F from *H*. *salinarum* and MMP0293 from *M*. *maripaludis*. It is an aldolase.

(6) **MJ1249** is homologous to OE1475F from *H*. *salinarum* and MMP0006 from *M*. *maripaludis*. It is believed to catalyze the second reaction in the AroAA biosynthesis, synthesizing DHQ.

(7) *Thermoproteus tenax*, crenarchaeon,

(8) *Pyrococcus furiosus*, euryarchaeon.

(9) GenBank accession number.

There is an error in the legend for Figure 3. The complete, correct Figure 3 legend is:

**Figure 3:**
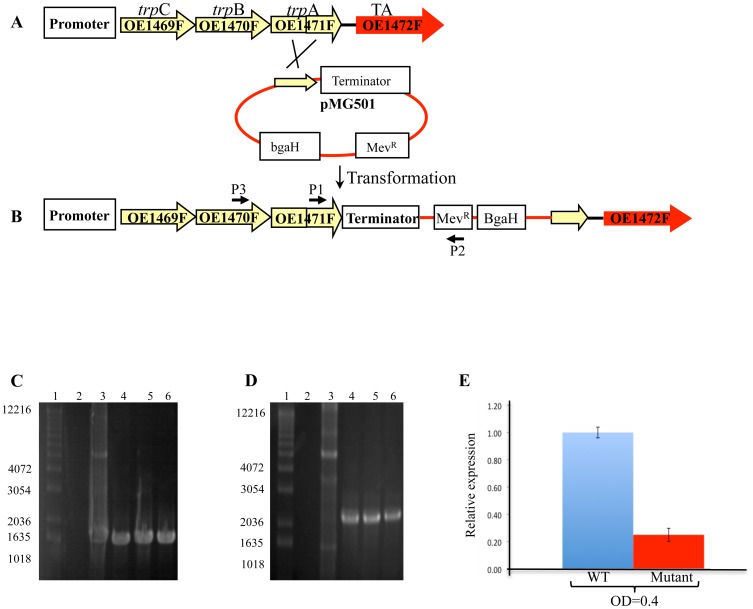
Schematic representations of a single cross-over event between the chromosomal DNA of *H. salinarum* R1 and plasmid pMG501. P1,P2 and P3 and their associated arrows indicate the relative positions and orientations of the primers used for PCR. After in-frame integration of plasmid pMG501, PCR was expected to generate a product in transformed cells but not for the WT. A, Illustration of WT *H. salinarum* genotype. B, illustration of the mutant genotype after incorporation of plasmid pMG501. C and D, Agarose gel electrophoresis of PCR products using primers P1 and P2, and primers P3 and P2, respectively. Lane 1: MW markers (in bp), lane2: Chromosomal DNA of R1, lane 3: plasmid pMG501 (control), lanes 4–6- transformed colonies. E, Relative expression of OE1472F transcription in mutant StopOE1472F (OE1471F::pMG501) grown in synthetic medium without AroAA to OD600nm  =  0.4. Results were obtained using RT-qPCR. See table S7 in file S1 for details of the primers used.

There are errors in the Supporting Information file and the legend. The line numbers were inadvertently left in File S1. The legend for Figure S2 is incorrect. Please see the complete, correct File S1 and legend here.

File S1 **Supporting files. Figure S1, Southern blot analysis of WT, stopEO1472F (A) and InsOE1475F (B).** A, Lane 1: size marker (in bp), lane2: SacI digested DNA from WT, lane 3: SacI digested DNA from StopOE1472F. B, Lane 1: size marker (in bp), lane2: NotI digested DNA from WT, lane 3: NotI digested DNA from InsOE1472F. **Figure S2, AroAA regulated CHY and HY ORFs that are closely adjacent, and possibly organized in operons. **Arrows show the relative positions and orientations of ORFs, but are not drawn to scale. The fold regulations are indicated below. The ORFs shown in panels A-D are described in Table 1. The ABC transporter ORFs shown in panel E is described in Table S2 in File S1. **Table S1, Formation of 3-dehydroshikimate (DHS) from 3-dehydroquinate (DHQ) under different conditions. Table S2, Masses detected by LC-MS after derivatization with NBHA. Table S3, Expression of AroAA-related genes and transport-related genes in H. salinarum R1 cells grown in synthetic medium without AroAA relative to synthetic medium with AroAA. Table S4, Strains used in this study. Table S5, The composition of the chemically defined medium, pH  =  7.0. Table S6, Plasmids used in this study. Table S7, List of oligonucleotides used in this study.**
(DOCX)
