# Supplementary material for: Correction: How Do Haloarchaea Synthesize Aromatic Amino Acids?
Source: PLoS One. 2014 Dec 19;9(12):e116355. doi: 10.1371/journal.pone.0116355 (PMC4272324; doi:10.1371/journal.pone.0116355)
Supplement: File S1 — Supporting files. Figure S1, Southern blot analysis of WT, stopEO1472F (A) and InsOE1475F (B). A, Lane 1: size marker (in bp), lane2: SacI digested DNA from WT, lane 3: SacI digested DNA from StopOE1472F. B, Lane 1: size marker (in bp), lane2: NotI digested DNA from WT, lane 3: NotI digested DNA from InsOE1472F. Figure S2, AroAA regulated CHY and HY ORFs that are closely adjacent, and possibly organized in operons. Arrows show the relative positions and orientations of ORFs, but are not drawn to scale. The fold regulations are indicated below. The ORFs shown in panels A-D are described in Table 1. The ABC transporter ORFs shown in panel E is described in Table S2 in File S1. Table S1, Formation of 3-dehydroshikimate (DHS) from 3-dehydroquinate (DHQ) under different conditions. Table S2, Masses detected by LC-MS after derivatization with NBHA. Table S3, Expression of AroAA-related genes and transport-related genes in H. salinarum R1 cells grown in synthetic medium without AroAA relative to synthetic medium with AroAA. Table S4, Strains used in this study. Table S5, The composition of the chemically defined medium, pH = 7.0. Table S6, Plasmids used in this study. Table S7, List of oligonucleotides used in this study. (DOCX) [file pone.0116355.s001.docx]

**Supporting Information**


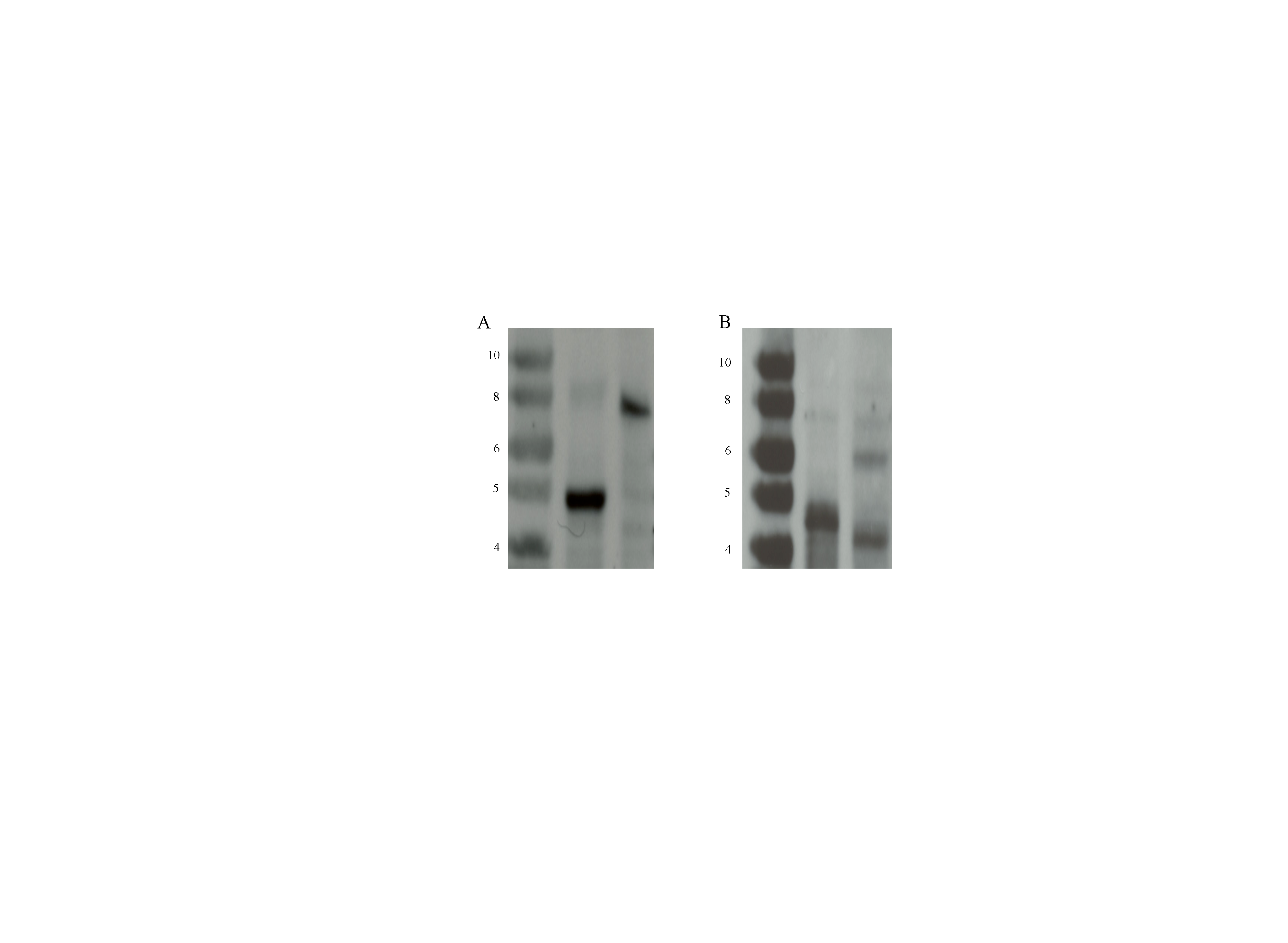


Fig. S1


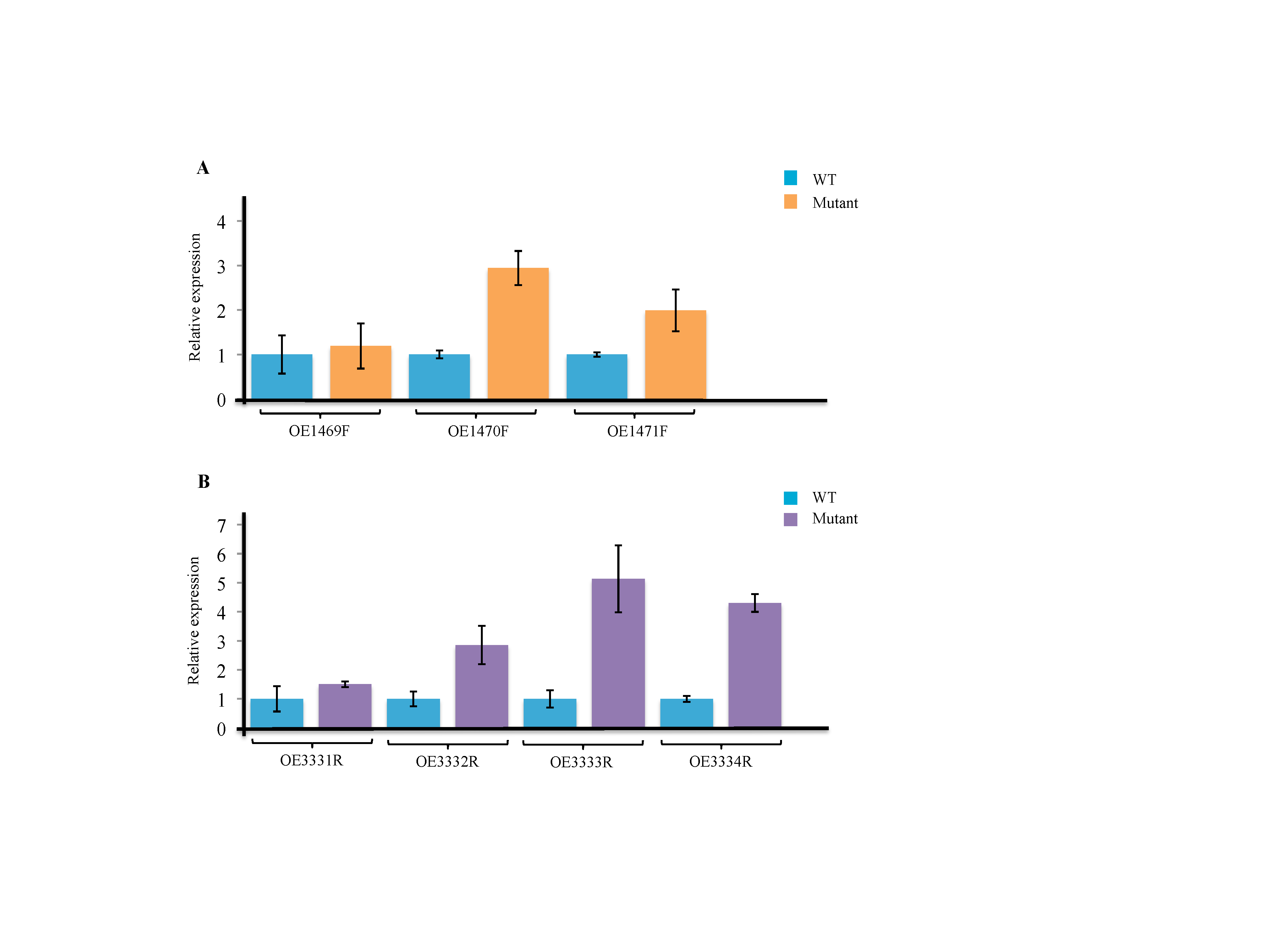


Fig. S2


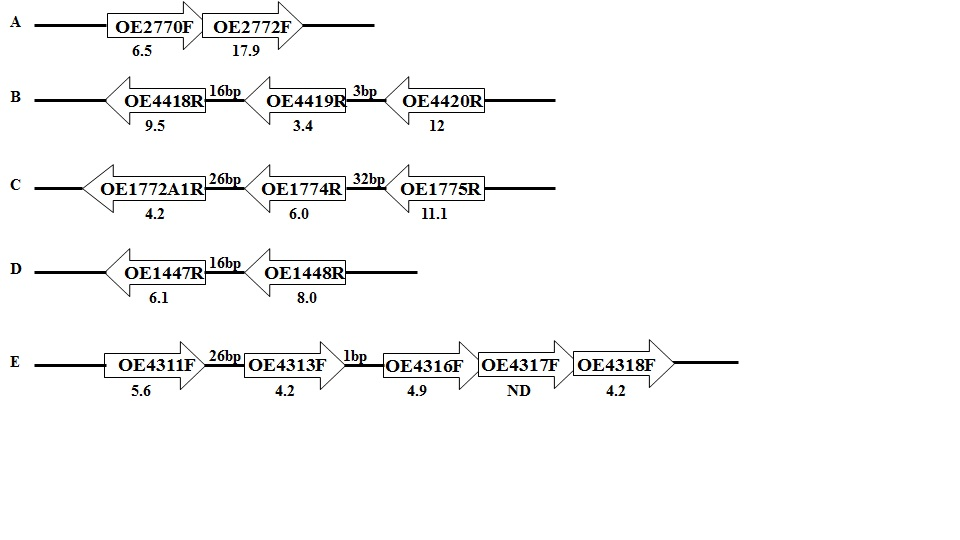


**Fig. S3**

**Table S1**: Formation of 3-dehydroshikimate (DHS) from 3-dehydroquinate (DHQ) under different conditions

| **^(1)^ Pre-treatment** | **^(2)^ Detection (%)** | |
| --- | --- | --- |
|  | **DHQ** | **DHS** |
| no pre-incubation | 99.6 | 0.4^(3)^ |
| 37°C, 1h | 97 | 3 |
| 37°C, 23h | 97 | 3 |
| 90°C, 1h in 0.1M HCl | 76 | 24 |

^(1)^ 2.5mM DHQ in 1.75M KCl+29mM Tris-HCl was pretreated before derivatization, according the conditions specified in the table.

^(2)^ Derivatization was carried out at 60°C for 1h, using 2mM NBHA [O-(4-nitrobenzyl) hydroxylamine hydrochloride] in MeOH. Derivatized compounds were separated on C18 columns using ACN. DHQ eluted after 15min and DHS after 20.2min. Percentages (%) were calculated based on the areas under the peaks detected at 254nm.

^(3)^ One cannot distinguish between the presence of DHS in DHQ and formation of DHS during the derivatization.

^(4)^ No difference was observed in the percent DHS after 1 hour and 23 hours incubation, indicating that equilibrium was achieved.

**Table S2**: Masses detected by LC-MS after derivatization with NBHA

|  | Formula | Exact Mass (g/mole) | Exact Mass after derivatization ^(1)^ (g/mole) | Mass detected by LC-MS ^(2)^ (g/mole) | RT (min) |
| --- | --- | --- | --- | --- | --- |
| ASA | C_4_H_7_O_3_N | 117.10 | 267.24 | 268.09 | 12.5 |
| DKFP | C_6_H_11_O_8_P | 242.12 | 542.39 | 543.11 ^(3)^ | 28.2 |
| F-1,6-P | C_6_H_14_O_12_P_2_ | 340.11 | 490.25 | 491.04 | 3.7 |
| DHAP, GAP | C_3_H_7_O_6_P | 170.05 | 320.19 | 321.05 | 14.5,  15.2 |
| DHQ | C_7_H_10_O_6_ | 190.15 | 340.29 | 341.09 | 14.8 |
| DHS ^(5)^ | C_7_H_8_O_5_ | 172.13 | 322.27 | 323.08 ^(4)^ | 20.1 |
| NBHA | C_7_H_8_N_2_O_3_ | 168.15 | -------- | 169.06 | 5.6 |

^(1)^-Calculated exact mass.

^(2)^-Calculated exact mass after derivatization and release of water molecules.

^(3)^-The exact mass as detected by LC-MS.

^(4)^- DKFP was derivatized twice and released of two molecules of water.

^(5)^- DHS was not used as a standard, but was identified by mass after the cleavage of DHQ by OE1477R.

**Table S3**: Expression of AroAA-related genes and transport-related genes in *H*. *salinarum* R1 cells grown in synthetic medium without AroAA relative to synthetic medium with AroAA.

| **ORF** | **Gene** | ^(1)^ **Predicted function** | ^(2)^ **Fold induction** | | |
| --- | --- | --- | --- | --- | --- |
|  |  |  | RT-PCR | Microarray | |
|  |  |  | OD_600nm_=0.2 | OD_600nm_=0.2 | OD_600nm_=0.58 |
| **Precursors DHQ** | | | | | |
| OE1472F | *fba*2 | 2-amino-3,7-dideoxy-D-threo-hept-6-ulosonate synthase | 12.5±0.7 | ND | ND |
| OE1475F | ------ | 3-dehydroquinate synthase (EC 4.2.3.4) type II | 1.5±1.2 | NS (0.96) | ND |
| **DHQ Chorismate** | | | | | |
| OE1477R | *aro*D | 3-dehydroquinate dehydratase (EC 4.2.1.10) | 2.3±1.7 | 1.6 | 1.3 |
| OE1565F | *aro*E | shikimate 5-dehydrogenase (EC 1.1.1.25) | NM | NS (1.35) | NS (2.56) |
| OE2785R | *aro*K | shikimate kinase (EC 2.7.1.71), archaeal-type | NM | 6.0 | 4.4 |
| OE2762R | *aro*A | 3-phosphoshikimate 1-carboxyvinyltransferase (EC 2.5.1.19) | MN | 3.6 | 2.1 |
| OE2761R | *aro*C | chorismate synthase (EC 4.2.3.5) | NM | 6.3 | 4.4 |
| **Chorismate Prephenate** | | | | | |
| OE2784R | *phe*A1 | chorismate mutase (EC 5.4.99.5) | NM | 2.0 | 3.8 |
| **Prephenate 4-hydroxyl phenylpyruvate** | | | | | |
| OE2770F | *tyr*A | prephenate dehydrogenase (EC 1.3.1.12) | NM | 6.5 | 2.7 |
| **Chorismate Tryptophan** | | | | | |
| OE3331R | *Trp*G1 | anthranilate synthase (EC 4.1.3.27) component II | NM | 2.1 | 1.4 |
| OE3332R | *Trp*E1 | anthranilate synthase (EC 4.1.3.27) component I | NM | 10 | 4.1 |
| OE3334R | *Trp*D1 | anthranilate phosphoribosyltransferase (EC 2.4.2.18) | NM | 6.2 | 4.5 |
| OE3333R | *Trp*F | phosphoribosylanthranilate Isomerase (EC 5.3.1.24) | NM | 8.5 | ND |
| OE1469F | *Trp*C | indole-3-glycerol-phosphate synthase (EC 4.1.1.48) | NM | 7.7 | ND |
| OE1470F | *Trp*B | tryptophan synthase (EC 4.2.1.20) beta subunit | NM | 8.9 | ND |
| OE1471F | *Trp*A | tryptophan synthase (EC 4.2.1.20) α subunit | NM | 6.7 | 3.6 |
| **Transport system of AroAA** | | | | | |
| OE2779F | *aro*P | amino acid transport protein (probable phenylalanine transport protein) | NM | 3.5 | 3.3 |
| **Sodium dependent transporters** | | | | | |
| OE3173F |  | sodium-dependent transporter | NM | 3.2 | 13.2 |
| OE5143F |  | sodium-dependent transporter | NM | 12.5 | ND |
| OE3511F | *pan*F | probable sodium/pantothenate symporter | NM | 4.5 | ND |
| **ABC-transporter** | | | | | |
| OE4311F | *dpp*A3 | ABC-type transport system periplasmic substrate-binding protein (probable substrate dipeptide/oligopeptide/nickel) | NM | 5.6 | ND |
| OE4313F | *dpp*B3 | ABC-type transport system permease protein (probable substrate : dipeptide/ oligopeptide/nickel) | NM | 4.2 | ND |
| OE4316F | *dpp*C3 | ABC-type transport system permease protein | NM | 4.9 | ND |
| OE4317F | *dpp*D3 | ABC-type transport system ATP-binding protein (probable substrate : dipeptide/oligopeptide/nickel) | NM | ND | ND |
| OE4318F | *dpp*F3 | ABC-type transport system ATP-binding protein (probable substrate :dipeptide/oligopeptide/nickel) | NM | 4.2 | 1.3 |
| OE4555F | *dpp*C2 | ABC-type transport system permease protein (probable substrate : dipeptide/ oligopeptide/nickel) | NM | 3.2 | 1.6 |
| OE3631F | *pot*A | ABC-type transport system ATP-binding protein (probable substrate: spermidine/putrescine) | NM | 2.9 | ND |
| **Hypothetical (HY) and conserved hypothetical (CHY) proteins** | | | | | |
| OE2776F | *Lrp*A2 | probable transcription regulator | NM | 14.9 |  |
| OE1775F | CHY |  | NM | 11.1 |  |
| OE4414 | CHY |  | NM | 3.8 |  |
| OE4412R |  | threonine synthase (EC 4.2.3.1) | NM | 3.2 |  |
| OE2770F | *tyr*A | prephenate dehydrogenase (EC 1.3.1.12)) | NM | 6.5 |  |
|  |  |  |  |  |  |

^(1)^ Functional predictions are taken from the genome annotation at [www.halolex.mpg.de](http://www.halolex.mpg.de).

^(2)^ The numerical data represent mean expression ratios in WT cells grown in medium without AroAA *vs*. cells grown in medium with AroAA. These were determined at two different cell densities. All fold changes had p values of ≤10^-3^, except where indicated.

^(3)^ Values in bold were determined by RT-qPCR. For all calculation the average-C_t_ (cycle threshold) of 2 replicate reactions per primer pair was used. The standard deviation was calculated from two biological repeats.

**ND**-not detected

**NS**- not statistically significant (p>0.01)

**NM**- not measured by RT-PCR.

Table S4: Strains used in this study

| Strain | Genotype or Phenotype | Source or reference |
| --- | --- | --- |
| *Halobacterium salinarum* R1 (DSM 671) | BR^+^, HR^+^, SRI^+^, SRII^+^, Car+, Rub^+^, Ret^+^, Vac^-^ | (Stoeckenius *et al*., 1979) |
| *Stop*OE1472F (OE1471F:: Terminator) | R1 derivative with OE1471F:: Terminator (No promoter/terminator for OE1472F), BR^+^, HR^+^, SRI^+^, SRII^+^, Car+, Rub^+^, Ret^+^, Vac^-^ | This study |
| *Ins*OE1475F (OE1475F::pMG501) | R1 derivative with OE1475F::pMG501, BR^+^, HR^+^, SRI^+^, SRII^+^, Car+, Rub^+^, Ret^+^, Vac^-^ | This study |
| ∆OE1477R | R1 derivative with ∆OE1477R, BR^+^, HR^+^, SRI^+^, SRII^+^, Car+, Rub^+^, Ret^+^, Vac^-^ | This study |

Table S5: The composition of the chemically defined medium, pH=7.0

| Compounds | Final concentration | Compounds | Final concentration |
| --- | --- | --- | --- |
| NaCl | 3.9M | L-Lys | 1.33 mM |
| MgSO_4_ x 7H_2_O | 78.8mM | L-Thr | 4.08 mM |
| KCl | 26 mM | L-Ser | 5.64 mM |
| KNO_3_ | 0.96 mM | L-Ala | 2.42 mM |
| Na_3_-citrate x 2H_2_0 | 1.65 mM | L-Gly | 0.98 mM |
| Glycerol | 109 mM | L-Pro | 0.88 mM |
| KH_2_PO_4_ | 0.41 mM | L-Asp | 2.92 mM |
| K_2_HPO_4_ x 3H_2_0 | 0.56 mM | L-Glu | 9 mM |
| CuSO_4_ x 5H_2_O | 0.19μM | L-Met | 1.3 mM |
| FeCl_2_ x 4H_2_O | 11.2μM | L-Val | 2.43 mM |
| MnSO_4_ x H_2_O | 1.7μM | **L-Tyr** | **1.1 mM** |
| ZnSO_4_ x 7H_2_O | 1.48μM | **L-Phe** | **1.1 mM** |
| Na_2_MoO_4_ x 2H_2_O | 0.96μM | **L-Trp** | **1.1 mM** |
| CoCl_2_ x 6H_2_0 | 0.98μM | Thiamine | 14.4μM |
| L-Arg | 2.23 mM | Folic acid | 11μM |
| L-Ile | 3.26 mM | Biotin | 2 μM |
| L-Leu | 5.92 mM |  |  |

**Table S6**: Plasmids used in this study

| Name | Relevant description | Source |
| --- | --- | --- |
| pMKK100 | 7.2Kbp; *H. salinarum* shuttle vector; Amp^R^, Mev^R^, *Bga*H | (Koch and Oesterhelt, 2005) |
| pMG501 | 7.777Kbp; *H. salinarum* shuttle vector; Amp^R^, Mev^R^, *Bga*H, **509bp of OE1471F (trpA)**, **modified terminator of FlgA**, parental plasmid -pMKK100 | This study |
| pMG601 | 7.729Kbp; *H. salinarum* shuttle vector; Amp^R^, Mev^R^, *Bga*H, **500bp of middle OE1475F**, parental plasmid -pMKK100 | This study |
| pMG700 | 7.213Kbp; *H*. *salinarum* shuttle vector; Amp^R^, Mev^R^, *Bga*H, **flanking regions of OE1477R**, parental plasmid -pMKK100 | This study |
| pET22b(+) | 5.49Kbp; *E. coli* expression vector; Amp^R^, 6xHis tag | Novogen |
| pMG560 | 6.165Kbp; *E. coli* expression vector; Amp^R^, **OE1472F**, 6xHis tag, parental plasmid - pET22b(+) | This study |
| pMG760 | 6.048Kbp; *E. coli* expression vector; Amp^R^, **OE1477R**, 6xHis tag, parental plasmid - pET22b(+) | This study |
| pMG860 | 6.159Kbp; *E. coli* expression vector; Amp^R^, **OE2019F**, 6xHis tag, parental plasmid - pET22b(+) | This study |

**Table S7**: List of oligonucleotides used in this study

| Primers for *in vivo* analysis of the mutants | Sequence |
| --- | --- |
| P1(*trp*A).for | GGCCGGGGATCCCGGCGTTCGTCTCCGCGGC |
| P2 (Mev).rev | GGCCGGGAACTCCTCGACGTGACGCCGTACGTCGTC |
| P3 (*trp*B).for | CGTGTCCGCC GGCCTCGACTACGCGGGGG |
| P4 (OE1475F).for | GGATCCGACGGCGAAGGCGACGGCAC |
| P5 (Mev).rev | GCGGCGCTGAAGGAGGCCGCGGAGGAG |
| P6.for | GGCCGGGGATCCTCGAGGGCGT CGCCACGTCC TG |
| P7 (OE1477R).for | GGCCGGGCTCGCCGCGTCAACCGGCGATC |
| P8.rev | CCGGCCAAGCTTCGCCACTTCGGGGAGGCCATCG |
| ∆OE1477R.F | GGCCGGGGATCCTCGAGGGCGT CGCCACGTCC TG |
| ∆OE1477R.R | CCGGCCCTGCAGACCCGGCTCACACGCGGCCAG |
| Primers for RT-PCR | Sequence |
| RT- L10ER.for | GTACCGGAAGATCGACAAGC |
| RT- L10ER.rev | GGAACAAGGCTGATCTGTACG |
| RT- OE1472F.for | ATCAGCACCGACGACAGAC |
| RT- OE1472F.rev | CGTTGAGGTGGACGATGTAG |
| RT- OE1475F.for | GAGGAAACCACGCTCGTC |
| RT- OE1475F.rev | CTCCTCGATGGTGGTGATG |
| RT- OE1477R.for | ACGACTACGACGGAGTGTTG |
| RT- OE1477R.rev | GTTGTGGTGTCTGTCGAACG |
| RT- OE1469F.for | CTCACCGAGCCACACCACTT |
| RT- OE1469F.rev | GGGTTCGGTCAACAGGAAGTC |
| RT- OE1470F.for | GTACGTCCCCGAGGTACTGATG |
| RT- OE1470F.rev | CTCGCGTTTGAGGTAGACGTCG |
| RT- OE1471F.for | GAGGGGACAACCATCCAGAA |
| RT- OE1471F.rev | GCCGTACTGGAACAGGAGGTT |
| RT- OE3331R.for | GATACCACTCCCTGGTCTGCAC |
| RT- OE3331R.rev | GTCCAGGAAGTTCCCGATGAC |
| RT- OE3332R.for | CTTGAGAGCGCCGAAAAGAC |
| RT- OE3332R.rev | GTCGTAGCCCACGAACGAGTAG |
| RT- OE3333R.for | TGACTCGCGTGAAAGTCTGC |
| RT- OE3333R.rev | GGCATCGTGACCAGGACACT |
| RT- OE3334R.for | GCCGAGGAGGTTGAAGATCGT |
| RT- OE3334R.rev | ACACGGCAACTACTCCGTCTC |
| RT- OE1160R.for | CTCGGTGAGGGCAACTACAAG |
| RT- OE1160R.rev | GCCTGTTCGACCTCACAGTAG |

漀
